# The Economic Lives of People with Disabilities in Vietnam

**DOI:** 10.1371/journal.pone.0133623

**Published:** 2015-07-21

**Authors:** Michael Palmer, Nora Groce, Daniel Mont, Oanh Hong Nguyen, Sophie Mitra

**Affiliations:** 1 Nossal Institute for Global Health, The University of Melbourne, Melbourne, Australia; 2 Division of Epidemiology and Public Health, University College of London, London, United Kingdom; 3 Inclusive Development Action (IDEA), Hanoi, Vietnam; 4 Department of Economics, Fordham University, New York, NY, United States of America; University of Perugia, ITALY

## Abstract

Through a series of focus group discussions conducted in northern and central Vietnam, this study gives voice to the lived economic experience of families with disabilities and how they manage the economic challenges associated with disability. The dynamic of low and unstable income combined with on-going health care and other disability-related costs gives rise to a range of coping mechanisms (borrowing, reducing and foregoing expenditures, drawing upon savings and substituting labour) that helps to maintain living standards in the short-run yet threatens the longer-term welfare of both the individual with disability and their household. Current social protection programs were reported as not accessible to all and while addressing some immediate economic costs of disability, do not successfully meet current needs nor accommodate wider barriers to availing benefits.

## Introduction

The conceptual pathways between disability and poverty are often noted. Poverty may increase the risk of disability through malnutrition, lack of access to safe drinking water, unsafe living and working conditions, or limited access to essential health services such as maternal health care or immunization [1]. In the other direction which forms the bulk of currently available empirical evidence, disability may lead to lower living standards and poverty for the household due to any range of physical, attitudinal or environmental barriers [2–5]. Understanding the specific cost factors that contribute to poverty and their dynamics remains poorly documented. Even less well understood is how families are coping with these costs and their implications for members of the household over time. This issue is particularly timely as governments grapple with the task of including persons with disabilities in national social protection programs in accordance with their obligations under the United Nations Convention on the Rights of Persons with Disabilities (CRPD), and other human rights initiatives.

This study was undertaken in Vietnam, a country with a large disabled population. According to census data, 7.8% of the Vietnamese population or 6.7 million people were living with a disability in 2009 [6]. This estimate likely underestimates the true extent of disability in the country. Vietnam is a signature member of the CRPD (22 October, 2007) and regional Incheon Strategy (2013–2022), both of which call for a range of social protection supports for people with disabilities [7, 8]. The government also recently passed a national disability law (July 2010) which outlines the right to state support across a range of sectors including welfare, health care and rehabilitation, education, vocational training, employment, transport, sports and entertainment [9]. Additionally, persons with disabilities are recognized as one of several social beneficiary groups eligible for state social protections, including a monthly cash transfer, non-contributory health insurance and education assistance [10–13].

Vietnamese people with disabilities experience higher rates of poverty relative to the wider Vietnamese population when accounting for the additional costs of disability [5, 14]. To gain a deeper understanding of the relationship between disability and poverty, this study adopts a qualitative approach to the questions of disability costs. The research aim is broadly to aid understanding about the lived economic experience of disability for the household and their expressed needs in light of recent efforts to extend formal social protections to persons with disabilities. Over the period November 2012 to September 2013, seven focus group discussions were held across northern and central Vietnam that addressed the following three key themes and questions: (i) What are the costs associated with disability for the household? (ii) What mechanisms do households use to cope with the costs of disability? (iii) How do available formal social protection supports perform against needs?

The following section, Section 2, provides a background on disability costs and their estimation. Section 3 describes the methodology and study design; Section 4 presents focus group interview results; Section 5 discusses the results; Section 6 addresses study limitations; and Section 7 concludes the paper with considerations for policy makers.

## On the Economic Costs of Disability

The economic costs associated with disability can be divided into indirect and direct costs [15]. The direct costs associated with disability include both additional expenditures on general items that any household may need, such as health care, transportation and food, as well as disability-specific costs such as rehabilitation and assistive devices, personal care assistance, and adaptations to housing and vehicles. Indirect costs do not represent an outlay of money but are foregone benefits (or opportunities) such as lost income of individuals or their caretakers who could not work or worked less due to disability. Lower earning capacity of the person with disability may result from limited work choice, anti-work incentives of government programs, longer amounts of time needed to complete tasks due to disability, or from educational, environmental, or social barriers to employment. Other family members may not be able to undertake paid work or may be restricted in the type, or number of hours of work they can perform due to care-giving responsibilities. Opportunity costs in foregone income for carers can be distinct from the actual number of hours spent care-giving. Having to care for a family member a few hours a day can preclude paid work, depending on the flexibility of scheduling. The full costs of care-giving might not be felt in economic terms until the future. For example, evidence shows that Vietnamese children with disabled adults in their household are significantly less likely to attend school [16].

Researchers have developed several different methodologies to estimate direct and indirect costs. We briefly summarize the key methodologies currently in use with a focus on direct cost estimation which forms the bulk of the literature. One approach is to directly measure consumption patterns in health and non-health expenditures between persons with and without disabilities whilst controlling for other sources of variation [2, 19, 20]. The limitation of this methodology is that studies are based on household expenditure survey data collected for the general population, where disability-specific expenditures are not itemized in the survey. Furthermore, expenditure item prices are assumed to be consistent across the population when a person with a disability may require more costly transport associated with the purchase due to taxi hire, for example. Moreover, costs are actual reported expenditures and thus require that items are available, accessible and affordable, which is particularly problematic in low- and middle-income country (LMIC) settings.

A recent and growing body of studies has adopted an indirect method to estimate disability costs known as the Standard of Living (SOL) approach [5, 14, 17, 18, 21–24]. Because it is an indirect method the SOL approach does not identify the specific items which contribute to additional costs but rather estimates costs overall and how they can vary across the disabled population (e.g. by impairment severity and type, life cycle, geographic location). The approach imputes as disability costs the extra income required for a person with disability to reach the standard of living of an equivalent person without disability. Most applications use material indicators of SOL, such as assets, and thus do not capture non-material aspects of welfare such as community participation. It is also assumed that each member of the household shares the same standard of living which is questionable in view of reports of households diverting resources away from disabled members in favour of non-disabled members [25, 26]. Furthermore, living standards are assumed to increase with income irrespective of disability status which is also questionable given the likely barriers faced by disabled people in converting income into life quality, referred by Amartya Sen as the ‘conversion handicap’ [27].

The above methods do not account for opportunity costs with respect to the loss of income due to disability and care-giving within the household–the ‘earning handicap’ as Sen puts it [27]. One straightforward approach in the literature is to compare head-count income poverty rates for disabled versus non-disabled households. This approach is problematic in LMIC contexts due to unreliability in the report of personal and household income associated with agrarian or informal labour markets and the high variability of income sources. Consequently, household consumption expenditure is commonly used as a proxy for permanent income and is divided by the number of household members to derive an individual income measure. Income is assumed to be equally distributed among disabled and non-disabled members of the household and therefore likely underestimates the extent of income poverty experienced by persons with disabilities. In a study of 15 LMICs, in only 3 countries were persons with disabilities found to have a higher rate of income poverty than persons without disabilities [2]. It is noted that the study finds a closer relationship between disability and multi-dimensional measures of poverty compared to proxy income based measures. The equivalisation of household income furthermore does not account adequately for the opportunity costs in income experienced by other household members due to care-giving responsibilities, similar to methods which attempt to estimate the income loss attributable to productivity gaps between disabled and non-disabled people [28].

In spite of the challenges in estimating the indirect costs among households with disabled members, the rate of income poverty is invariably increased when the direct costs of disability are taken into account [5, 17, 23, 24]. The SOL approach has been used to expand the concept of poverty beyond conventional income or consumption based constructs. The rationale is that standard poverty lines which assume that the minimum basket of resources encapsulated in the poverty threshold is sufficient to meet basic needs are insufficient for persons with disabilities because of the additional disability-related expenses. By failing to take account of these costs, standard poverty lines routinely underestimate the degree of income deprivation among the disabled population. For example, in Vietnam, the extra costs are estimated at 11.5% of income. Once these extra costs are taken into account, the poverty rate of families with disabled people increases from 17.6% to 22.3% [5].

Prevailing quantitative methodologies fail to provide a full picture of disability costs, the inter-connecting relationships of costs and their effect on the individual and household. The purchase of a wheelchair, for example, may reduce indirect costs on the household by leading to improved scores in independence (as measured by a care-giving support scale) and levels of employment and income [29]. How individuals and families negotiate competing economic demands, particularly when money and resources are most limited, are furthermore not explored. A small number of studies have recently examined the impact of household coping mechanisms [30–33]. The studies are quantitative in nature and thus limited by the data under study. Disability measures are imperfect and narrow in scope (e.g. the number of days unable to perform regular activities, such as employment or education, due to illness or injury in the past month), as is the range of coping mechanisms (reallocation of expenditures, loans and sale of assets). Like the majority of disability cost estimations, the time under study (2–4 years) is also unsuited to assess the longer-term impact on household welfare which is problematic since disability is often a long term or life-long condition as distinct from more transient health conditions.

For the above reasons, we undertook a qualitative study of disability costs in Vietnam. Using a series of focus groups, we give voice to the lived economic experience of families with disabilities and how they balance competing demands on household incomes. In light of the recent efforts on the part of the Vietnamese government to protect and promote the rights of persons with disabilities, we further asked study participants whether and how current social protection schemes contribute to improving the economic status of the household. Our approach suffers from the same limitations as other subjective approaches that rely upon expert panels, or disabled persons themselves, to assess disability costs [17]. Reported costs will depend upon the nature and severity of impairment, as well as other individual, household, and community level factors, and there exists no counterfactual from which to level disability costs.

## Methodology and Study Design

Ethical approval for the study was obtained from the University College of London (No.:1967/005). Because of low literacy levels in the population under study, informed verbal rather than written consent was anticipated and applied for as part of our ethical approval process. Verbal consent was approved by the UCL Institutional Review Board and obtained from all study participants in accordance with verbal consent procedures and capacity to consent as determined by legally authorized disabled people’s organizations. Parental or next of kin consent was obtained for persons under age 18 and all consent was documented on record.

A pilot focus group discussion (5 participants) was conducted in Da Nang city in November 2012 by two of the authors of this paper (MP, DM) at the central region office of Vietnam Association for the Handicapped (VNAH). Building on this, in September 2013, a series of six focus groups (72 participants) were organized in and around Hanoi in collaboration with research partners Inclusive Development Action (IDEA), a disabled people’s association, and the University of Labour and Social Affairs (ULSA), the university arm of the Ministry of Labour, Invalids and Social Affairs which is responsible for the implementation of the national disability law. The six focus groups in the northern location were evenly divided into an urban ward in Hanoi (Đống Đa) and a semi-urban district outside of Hanoi (Thanh Trì) because we believed that economic costs and concerns might vary in these different localities e.g. labour markets and services (health care, care-giving, public transportation) are more limited in semi-urban and rural areas, for example.

All participants of the study were identified through local disabled people’s associations since we wanted selection to be based upon people’s own understanding of disability in their lived environment rather than legal or survey definitions so as to provide a nuanced understanding of how the heterogeneous nature of disability impacts the economic lives of families. Among the 77 participants recruited, the vast majority (68) were persons living with a disability of working age (range 17–65 years) as we wanted to better understand the economic priorities and concerns of disabled adults from their own perspective. The other nine participants were the parental guardians of persons with disabilities, including mothers or grandmothers of four children with disabilities (aged 5–7 years) who spoke specifically to the costs associated with raising a child with a disability.

Where possible, participants were selected evenly on the basis of disability type and gender. Thirty-nine of the 77 participants were female (51%). A breakdown of the participants by disability type and location is provided in Table 1. Over half of the participants had physical disabilities and approximately one third had sensory disabilities, with the remainder having mental disabilities as the main disability. Mental health disabilities were divided according to Vietnamese terminologies: trntalệ chậm phát triển (learning or developmental disabilities), thiếu năng trí tuệ (intellectual disabilities), and hội chứng tự kỷ (austism spectrum disorder). Autism is classified as distinct from mental health disabilities in Vietnam [34].

**Table 1:** Focus group participants by disability domain and interview location.

	Hanoi		Da Nang	Total
	Dong Da	Thanh Tri		
**Mobility**	**20**	**18**	**5**	**43**
**Visual**	**4**	**9**	**0**	**13**
**Hearing**	**9**	**0**	**0**	**9**
**Speaking**	**1**	**0**	**0**	**1**
**Intellectual**	**2**	**2**	**0**	**4**
**Developmental**	**2**	**2**	**0**	**4**
**Autism**	**1**	**2**	**0**	**3**
**Total**	**39**	**33**	**5**	**77**

Where possible, focus groups were organized to elicit information from participants within the same disability type so as to make participants feel more at ease and foster a rich discussion. This included a deaf group (with sign interpreter) in Đống Đa, a group of individuals who were blind or vision impaired in Thanh Trì, and a group with physical disabilities in each of the three locations. Two groups contained a mix of people with different disabilities in the northern locations.

Questions for the focus groups were developed from reviews of the literature and institutional knowledge of the policy context in Vietnam as well as previous in-country fieldwork on disability by two of the co-authors (DM, MP). To ensure clarity and cultural appropriateness, the questions were reviewed by research partners. Focus groups were conducted by a research team member from IDEA and ULSA, with one additional team member observing and taking notes. The discussions were digitally recorded, transcribed and thematic content analysis was performed. The key themes and quotes that emerged were then summarized and translated into English. Four key themes and linked sub-themes were identified from the discussions and are presented in the following section.

## Findings

### Indirect costs

#### Indirect costs for persons with disabilities

The vast majority (88%) of disabled working-aged participants in our study were working. Due to the existence of multiple barriers in entering the formal labor market, persons reported that they often had to create their own jobs in the informal sector. Jobs were typically low-skilled such as small shop vendor, tailor, laborer, agricultural worker, handicraft worker or masseuse. The income derived from these jobs was low and unstable and often insufficient to meet expenditure needs:

There are some jobs that I can do but the income is so low that it does not meet my economic needs.

Sometimes I have work, sometimes I don’t. We are a family of four with two children going to school. Because of unstable income and school fees are very high, economic challenges are always greatest for our family.

Limited education and lack of professional training certificates were repeatedly cited as barriers to entering the work force. As one young man with a hearing impairment said: “Most of us have not finished secondary school… in today’s competitive labour market this is very difficult.” Due to a lack of sign-interpreter supports in the classroom, and accessibility to hearing aids, it was difficult for deaf people to progress beyond primary school.

But even when people with disabilities had achieved higher qualifications, discrimination by employers was perceived as the greatest barrier to securing paid employment.

When arriving at an interview, I was told that they had found someone already. They said this maybe because they saw me as having difficulties in speaking and in weak health.

I studied pharmacy but I cannot find work as a pharmacy assistant. Nobody will employ me. I want to open my own small pharmacy shop but I do not have the capital.

I tried to create conditions for him (son) to continue studying at technical college in IT and he graduated with average marks. But when he applies for jobs, employers only encourage him, they never accept him.

Another barrier identified was that persons with disabilities repeatedly reported that they were anxious to work, but family members often did not believe in the self-reliance and work capability of members with disabilities, reflecting broader restrictive societal views on what persons with disabilities can do.

Teaching him how to take care of himself and how to maintain personal hygiene are already high hopes…I don’t dare hope that he is capable of working.

People look down on us. When people see me, they ask me whether I could do anything and I tell them that I can do whatever you can do. You do it your way and I do it my way.

Hearing and speaking people always think our (sign) language is a joke, a humorous thing to make people laugh. But I don’t think so. I think that is wrong.

#### Indirect costs for non-disabled household members

The majority of study participants did not have high carer support needs but often required transport or had to be accompanied by a family member when out and about in the community. The need for such support was an ongoing drain on carers’ time and infringes upon their work schedules. For these study participants, care-giving duties were incorporated into the daily domestic duties of other family members.

It is very difficult to get permission to be late 2 hours for work to take my child to acupuncture. I must work over lunch and stay back late. I don’t dare ask often because I am afraid that I will lose my job.

I am retired but I cannot do relief teaching and my monthly pension is low, 3 million (dong) per month, because I have to spend all of my time taking care of my son, taking him to school and to the hospital for check-ups.

In some cases the indirect opportunity costs of care-giving responsibilities were substantial, resulting in the partial or complete loss of income.

When I found out that my child had autism from the age of three, I quit my job to take care of him carefully.

I retired early to take care of my 30 year old son who has cerebral palsy and my grand child who has autism and hyper activism.

### Direct disability costs

Respondents reported a series of disability-related expenses clustered around several key topics that consistently proved a drain on household budgets. Hospitalization, school fees, and purchase of an assistive device or adaptation of a vehicle were large periodic expenses for some participants. More commonly, it was the relatively smaller on-going expenses associated with disability including medication, rehabilitation, travel, and private tuition that accumulated over time and proved to be costly for families.

#### Health care

Health care and related costs were consistently identified as the most burdensome. Episodic costs associated with hospital visits were compounded by costs associated with the hiring of a private taxi or specialized vehicle, as well as accommodation costs for accompanying family members to take care of the person with disability whilst in hospital which is common due to limited nursing staff. Medication and rehabilitation were considerable on-going expense for many families, with several families reporting monthly medication expenses in excess of 1 million dong or over one-third of the minimum wage (2.7 million dong in greater Hanoi [35]; 1 USD ≈ 22,000 dong). As one blind respondent said, “Every time my eyes are sore I must buy some medications… it is not a small amount from my little income.”

#### Rehabilitation

Rehabilitation services are currently limited to basic physical rehabilitation services with orthopedic, speech and language, and occupational therapies in short supply across the country. Such services are for the most part only available at provincial level hospitals with a small number of beds relative to needs. In the province of Bac Ninh, for example, there are 200 hospital beds reserved for rehabilitation services including 150 beds at the provincial rehabilitation hospital and 50 beds in the rehabilitation wing of the main provincial hospital. Approximately half of the provinces in Vietnam have a rehabilitation hospital and each has a rehabilitation ward in the provincial level hospital. To claim rehabilitation services under insurance one must obtain a referral letter from the registered health facility or else incur a co-payment of 50%. As one respondent put it, “to receive the quality care you must have a letter of referral but to obtain this you need to have a good relationship with the doctor. If you do not, then it is difficult to get the letter.” A community-based rehabilitation (CBR) model is being developed across the country but is currently on a small scale. The primary rehabilitation services accessed by participants in our study were acupuncture and acupressure services, available in traditional medicine departments of district level hospitals and private outpatient clinics.

#### Assistive devices

The most common assistive devices identified by participants were basic and included walking sticks, crutches and spectacles. There is a large unmet need for assistive devices, particularly prostheses, wheelchairs and hearing devices. Such ‘modern’assistive devices are not easily accessed and are prohibitively expensive:

It’s very difficult for me to pay for general medical expenses and I am incapable of buying modern hearing aid devices.

Mobile phones have to be designed specifically for us, not every blind person can find or afford them.

Assistive devices also entailed periodic costs for repair and replacement. One participant from Da Nang reported prostheses purchases totaling over 6 million dong or 2.5 months equivalent minimum income every three years [35]:

I have to buy a walking stick to support me. It costs around 120,000 dong or 150,000 dong and it needs to be replaced every six months.

Every three years I must replace the prosthetic pieces at a cost of 2.1 million dong for the groin piece and 4 million for the knee piece. In addition, I must spend approximately 800,000 dong per year on bandages and shoes for my disability. I have a health insurance card but I must pay everything myself.

#### Transportation

Outside of health-related expenses, transportation costs for taxis or motorcycle taxis were an on-going concern for many participants, over half of whom had mobility disabilities. Costs are attributable, to some extent, to difficulties in accessing public buses, lack of assistive device technology and motorized tricycles that would increase ability to travel within and beyond their communities. As a consequence, the ability to travel to work, to do errands or to seek medical care was limited by affordability of transportation. Family members were often relied on for transport, which carries a further and often forgotten cost for the household.

I had an accident recently. It affected my legs so I have to use a motorbike taxi to get around. However, it’s quite expensive. It costs about 10,000 dong per kilometer.

I would like to buy a three wheel motorbike but they are very expensive, it costs an additional 4–5 million dong to adapt a two wheel motorbike. Instead, I must rely on my brother to transport me around on his motorbike but I feel like a burden to him.

#### Education

Focus group participants routinely reported economic limitations based on lack of education and vocational training earlier in life. The small group of mothers and grandmothers of disabled children who participated in focus groups shed additional light on this, reporting barriers to entry into public schools for children with disabilities. Because many disabled persons continue to be denied access to public schools many families are forced to seek alternative education for their disabled child through special schools or private home tutors. These are associated with significantly higher fees that are not affordable by all households, and represent a significant drain on household resources over a period of years.

I was faced with lots of difficulties when trying to enrol my child into a public school. Private schools or special schools have high fees, not all households can afford to go to such schools.

My child has autism and cannot join public schools with normal fees. She has to go to special schools or have a private home tutor which is much more expensive. It costs about 4–5 million (dong) per month.

I must hire a private tutor for my child which costs 200,000 dong per hour. Every month, I must pay about 3 million dong for my daughter’s medicine, reflexology and her school fees.

The topic of vocational education/training was a greater concern in the focus groups due to the working age of most participants. Here again, few people had benefited from public vocational training programs. Instead they had to seek private vocational training (where available) which were reported as limited in scope and in some cases, prohibitively expensive. One focus group participant gave as an example:

There are not many (training) choices for us, even to complete the massage certificate costs 3 million dong which is not affordable to everyone.

#### Household coping mechanisms

Borrowing was a primary mechanism through which families coped with the major costs associated with disability. The majority of participants were able to access finance from state subsidized money lenders or the local disabled people’s organization at a low rate of interest to start a business to generate more permanent income. Interest-free loans were secured from relatives and friends to pay for periodic medical or school fees. Difficulties in securing credit from formal money lenders was also mentioned:

If a person with a disability is not affiliated with an association (disabled person’s association), nobody dares to lend him money, even as little as 1 million dong.

Other coping mechanisms reported were drawing upon private savings and substituting labour within the household, but these were less common. Households commonly had few savings to withdraw (“How could I possibly have savings?”). Labour substitution within the household related mainly to off-farm seasonal work in low-skilled manual jobs which were characterized by low and irregular income therefore supplemented household income only marginally.

I cannot do any work, I have a big family with three children going to school. My wife struggles alone to raise the family. Our main income is from agriculture. Between crops, my wife has to find other manual jobs to earn more income. However, income from these sources is low and unstable.

Individuals and families routinely reported cutting-back on, or foregoing, disability-related expenditures.

The fees for every time I have an operation are very expensive and I must pay everything. I have had four operations already and for two years I have not been to see a doctor after the last operation because I am afraid that I will have to have another operation and I do not yet have the money.

…the medicine for his brain is 2 million (dong) for one time so I only buy it occasionally.

I must pay 4 million dong per month for my child’s school fees…after one year I had ran out of money so I sent him/her to back to the farm.

I would like to buy an electric bicycle so as to commute more conveniently and make me less tired but these days I just use my old bicycle.

### Social Protection

With the passing of the national disability law in 2010, Vietnam consolidated a range of social protection supports for persons with disabilities across the country [9, 36]. Persons with profound or severe disabilities are entitled to monthly income support, health insurance, education assistance, and public bus fare and other travel exemptions. Households with profoundly disabled members are also entitled to an additional monthly caregiver support allowance. Any person with a disability, identified under the law, is eligible for loans with preferential interest rates from the Social Policy Bank.

Determination of who is eligible for social protection supports is made by commune committees comprising a president, local Government and union representatives using a disability assessment tool [37]. Over half (58%) of our study participants were receiving income support and health insurance, either through the disabled person’s association or local commune/ward committee. People either received all available social assistance or nothing as one respondent said: “My husband and I pay everything ourselves; there are no outside supports.”

Social protection supports, in general, were gladly received but were not consistent with reported needs. The most successful program, as judged by respondents, was the social credit program where applicants could apply for a low-interest loan for business development and job creation activities. Bus fare waivers and education assistance were viewed as the least successful programs due to problems in accessing public buses and schools. Specific comments on each program included:

#### Monthly income support

According to our informants, the amount of money currently available through monthly payments did not make a major difference in the expenses of recipient households. Amounts varied from a minimum of 360,000 dong for a single person payment to a maximum of 720,000 dong for a household with an additional carer allowance. These sums constituted between 13–26% of the monthly minimum wage in the areas under study. As one recipient put it, “the amount is not even enough to buy rice (for the family).”

Other concerns raised were with complex application procedures and the fact that not all persons with disabilities were eligible for monthly support, including children.

I am currently preparing my application file, but the paperwork and administration is excessive/complicated so I am having difficulties. I don’t know when I will receive the money.

The wish of every person with a disability is to receive the income support to ease some of their economic difficulties but in reality this is not the case.

#### Health insurance

The majority of recipients found the non-contributory health insurance programme and the health insurance card useful whereas others reported it was “virtually unused” due to out-of-pocket payments, perfunctory examinations, long waiting times and administrative procedures. A repeated concern was that insurance did not cover all of the costs of required services, equipment and medication with some respondents paying a significant proportion of the total health care costs out-of-pocket.

I have sleep apnea and it is not included in the list of exempted conditions hence I must buy all the necessary equipment.

My child needs acupressure treatment. It costs about 10 million (per year) however insurance only covers 30%, the remaining 70% is a lot for us to cover.

My eyes often get sore and need medication. Health insurance covers a part but many types of medication are not included on the list of exempted or discounted medications.

Costs associated with assistive devices or travel to health facilities were not covered under insurance, and focus group members reported that often local doctors did not give the referrals needed for rehabilitation services available at provincial hospitals, which imposed higher costs on the families pursuing treatment. As was true of the Monthly Income Support programme, a widely voiced concern was that not all persons with disabilities were able to receive non-contributory health insurance.

Currently, only persons that are identified as social beneficiaries and eligible for the monthly income support can receive the free health insurance card. I do not think that this is fair as all people with disabilities have health care needs.

#### Public bus fare waivers

Few persons with disabilities reported being able to take advantage of the public bus fare waiver. Part of this was due to difficulties in getting on and off buses without assistance, particularly for people with mobility impairments. As one person stated: “catching a bus on my own is nearly impossible for me.” Many bus drivers and members of the public had little or no awareness of how to assist people with disabilities in getting on or off the bus. The result was, as another physically disabled person stated, “when catching a bus, I am often left behind. There are times I could not get on a bus even after two or three hours waiting.”

#### Low-interest loans

Loans sourced from the State Social Bank for business development or job creation activities were adjudged as suitably flexible to the needs of persons with disabilities. The loan conditions included a maximum amount of 20 million dong for a term of two years at a monthly rate of interest of 3.6% paid in monthly or tri-monthly instalments.

I think it (social credit program) is good. The interest rate is low and you do not need collateral, which suits us as most of us are poor. The application procedures are simple, not much paperwork. And you have options when you can pay back, this also suits us because sometimes we have money (to pay the loan), sometimes we don’t.

It is noted, however, that some respondents reported using the loan for other than its intended purpose, instead being used to meet pressing medical expenses or to purchase an assistive device.

#### Education assistance

Because members of our focus groups were adults, few study participants had benefited from the 2010 Government decree on public school fee exemptions for poor children with disabilities, [13]. One issue raised by a number of informants, however, was that public schools were still not inclusive for all children with disabilities while special schools, which were prohibitively expensive even for non-poor families, were not subject to fee discounts. Vocational training was also consistently recognized as a large unmet need. As one participant noted: “what we blind people really need is programs that train us in professional job skills that are suitable to us and can earn a good salary, such as computing and accounting.”

## Discussion

Overall, the evidence from quantitative studies thus far points toward individuals with disability having sizable extra costs. These include both costs associated with opportunities foregone and those directly associated with disability. Quantitative methods tell us little explicitly about how costs interact and how individuals and families attempt to balance or manage these extra costs and what the longer-term impact on their welfare is. The qualitative approach adopted in this paper offers further insight into these issues in the context of Vietnam, a low-middle income country with a relatively advanced legal and institutional framework for the provision of social protection support for persons with disabilities. We explore the economic costs of disability and how current state supports reflect and address the expressed economic needs of households.

With respect to the costs of disability, our study results points to a dynamic of low and unstable income combined with the extra direct costs of disability as the cause of economic difficulty for individuals and families. The majority of our disabled participants were working yet due to multiple barriers in entering the formal labour market, had to create their own employment in the informal labour market. The level of income generated through these informal jobs was not sufficient or stable enough to meet the costs associated with their disability. Findings are reflected in national living standards data where among persons with disabilities that were working (44%), the overwhelming majority were working the informal sector either as farmers or self-employees (87%) (our calculation using the 2006 Vietnam Household Living Standards Survey (VHLSS) and high disability threshold as defined in [5]).

Health care and related-costs were particularly burdensome for individuals and families. A major concern reported by our study participants were medication and travel costs associated with health facility visits. Once again, findings are consistent with national living standards data where these two cost items comprise 64% and 75% of total inpatient- and outpatient-related costs, respectively, for persons with disabilities [38]. Aside from costs associated with health care facility visits, households reported a variety of on-going expenses associated with disability, including medication, rehabilitation, travel and private tuition. The on-going costs concerned people most because they affected people’s daily life and decisions. All direct costs reported occurred throughout the life cycle (all ages) hence they are expected to have an impact on today’s economic wellbeing, but also a cumulative impact in the long term.

We found significant indirect costs for many households associated with caregiving, which included time spent transporting the family member with disabilities, and in accompanying them on health facility visits. Costs associated with care-giving were greatest for persons with disabilities that were not working, which further compounded income deprivation for these families. This was not the case for the majority of our study participants whose care-giving was incorporated into the daily domestic duties of other household members. People with high care needs were presumably less able to attend our focus groups. Another issue is that the majority of people in the focus groups were disabled adults and thus may be unaware of choices made by carers at some earlier point in their lives where opportunities or options for other types of work were forgone.

The costs associated with disability invoked a range of coping mechanisms among households. Borrowing was a primary mechanism through which families coped, consistent with quantitative findings on health and disability shocks from Vietnam [30, 32]. The majority of participants were able to access cheap finance, which may be a reflection of the higher levels of social capital of the group under study. However, difficulties in gaining access to formal credit organizations were mentioned, which is a notable concern for people without access to cheaper forms of credit or informal networks such as ethnic minority persons with disabilities in remote areas of central Vietnam who draw upon high-interest loans and sold land to pay for medical costs [26]. Other cited coping mechanisms, including drawing upon private savings and substituting labour within the household, are supported by other disability research from Vietnam [26, 32].

Quantitative findings indicate that such coping mechanisms help Vietnamese households to maintain consumption in the short to medium term [32]. However, they are based upon actual expenditures and ignore the opportunity costs to the household. Foregoing expenditures on education, health care, medication, rehabilitation, assistive devices and specialized vehicles for family members with disabilities were all reported in our study due to a range of reasons including lack of availability, accessibility or affordability. Forgoing critical expenditures for persons with disabilities deprive them of the opportunity to participate more fully in productive, domestic and community life, which has spill-over effects on the lives of other family members.

Meeting school fees of children on a single income when one parent was not working due to disability was identified as a particular challenge in these focus groups, comparable with findings from India [25]. Furthermore, quantitative findings from Vietnam reveal a reduction in household education expenditures in the event of disability or illness [32], and that non-disabled Vietnamese children with disabled adults in their household are significantly less likely to attend school [16]. This is expected to carry lasting inter-generational effects to household welfare as returns to human capital investment in LMICs are high [39]. These findings have relevance to policy makers since typically, as in Vietnam, non-disabled children of persons with severe disabilities are not entitled to school fee exemptions.

Whilst the proportion of participants in this study receiving Government supports was higher than the national average, likely due to the political connectedness of the group, many households did not receive any social assistance supports. In 2006, about one-third and one-half of Vietnamese persons with a high disability threshold received monthly income support and health insurance, respectively (our calculations using VHLSS 2006). One concern consistently raised by focus group members was that not all persons with disabilities were eligible for social protection supports. Currently, under the national disability law only persons classified as having severe or profound disabilities—persons with disabilities who are unable to perform some or any basic activities of daily living such as walking, dressing, and personal hygiene—are entitled to social protection supports [36]. This likely only captures persons in the highest disability threshold. The findings from this and other studies on disability and poverty in Vietnam [5, 14, 38] suggest that persons with mild/moderate disabilities, as defined under the law, also have high needs for social protection support: “persons with impairment which suffer functional limitation and difficulties in activities of daily living, work, or study” [9].

Another predominant theme among focus group members was the lack of the current social assistance programmes to provide adequate funding for persons with disabilities to meet their economic needs. Cash transfer amounts were low by income replacement standards, and health insurance did not offer financial protection against all of the costs of care consistent with quantitative findings from Vietnam [20, 40]. Assistive technology is a notable omission from insurance entitlements and a concern given the low level of usage and high cost of particular assistive technology found in this and in other studies in Vietnam [41]. Rehabilitation services are a recent addition to the list of claimable insurance services in Vietnam yet accessibility remains low due to supply side barriers, consistent with findings from a government survey of seven communes in 2011 where four percent of persons with disabilities reported accessing formal rehabilitation services [42]. Public bus and school benefits are also limited by accessibility barriers, which result in added direct and indirect costs for the household. Parents (and siblings) of children currently denied school attendance often either pay for prohibitively expensive private education or tutoring, or take additional time out of their days to provide some substitute educational efforts. Similar concerns are voiced among parents of children living with autism spectrum disorder in Hanoi [34].

It is important therefore not only that benefits are commensurate with needs but sufficient accommodations are made so that persons can avail themselves of benefits. The latter point is supported by quantitative evidence of improved district level health services, roads, and other indicators of good infrastructure lessening the link between disability and poverty in Vietnam [43]. A positive and unexpected finding from this research relates to the low-interest loan program, which likely has poverty alleviation effects in view of the difficulties that persons with disabilities face in securing paid employment and in accessing other microfinance programmes [44]. The longer-term implication of this indebtedness for the household remains unclear.

### Study limitations

We acknowledge an inherent bias in the recruitment of our sample in that not all persons and families with disabilities in Vietnam are associated with disabled people’s organizations from which participants were recruited. We did not find any significant disparities in the lived economic experience of persons, and their families, by focus group location (semi-urban vs. urban), which may reflect the fact that the semi-urban district was a part of greater Hanoi or that participants in the urban ward were not able to take advantage of increased formal employment opportunities. It is noted, however, that many of the costs and barriers reported in this study are similar to those found among youth ethnic minority people with disabilities, and their families, in a remote area of central Vietnam [26]. Finally, the experiences and voices of persons with intellectual and mental health disabilities are less represented in this study due to difficulties in recruiting these persons as they were under-represented within the local disabled people’s organizations.

## Conclusion

The economic lives of persons with disabilities and their families are distinct to other population groups with respect to the long-term intra-family effects. However, comparably little is understood on the economics of the population with disabilities. The majority of our study participants were working yet subject to economic hardship through the combination of low and unstable income with the additional burden of the extra costs of disability. The coping mechanisms commonly adopted by families to deal with these costs including borrowing and reducing and foregoing certain expenditures impeded their ability to invest in key expenditures for the individual with disabilities and other household members, threatening their economic welfare over the longer term. These findings show that there is a big gap between the goods and services used and those required thus drawing attention to the limitation of prevailing quantitative methods in the estimation of disability costs focused on the goods and services used.

Relative to other countries of equal or even greater level of development, Vietnam offers a strong suite of social protection supports for persons with disabilities. However, large challenges remain in extending supports to all those in need and in providing benefits commensurate with needs while providing sufficient accommodations in the environment so that persons can fully avail themselves of these benefits. One novel finding from this research is that low-interest loans may provide an important short-term safety net for persons with disabilities, and their families in Vietnam and perhaps more generally in countries where, social protection systems are in their infancy.

A conclusion based on these focus groups is that co-ordination across government is required to build a strong and integrated system to support the economic integration of persons with disabilities, which remains a considerable challenge for Vietnam and for LMICs in general. In addition, as in many countries, broader attitudinal barriers within the family and the wider community are significant barriers to improving the economic standing of persons with disabilities in Vietnam. Improving attitudes around the capabilities of persons with disabilities within the community may significantly reduce several of the costs associated with disability for the individual and the household.

## Supporting Information

S1 Data (DOC)
