# Supplementary material for: The Economic Lives of People with Disabilities in Vietnam
Source: PLoS One. 2015 Jul 21;10(7):e0133623. doi: 10.1371/journal.pone.0133623 (PMC4510056; doi:10.1371/journal.pone.0133623)
Supplement: S1 Data — (DOC) [file pone.0133623.s001.doc]

**BÁO CÁO KẾT QUẢ PHỎNG VẤN**

**Thời gian thực hiện:** ngày 18-19/9/2013

**Kết quả buổi phỏng vấn:**

1. **Thông tin chung về nhóm đối tượng được phỏng vấn**

- Dạng khuyết tật: khuyết tật vận động, trong đó có 18 người khuyết tật ở chân chiếm tỷ lệ cao với 75%), tiếp theo đó là khuyết tật ở tay chiếm 20% và khuyết tật cột sống chiếm 4%.
- Số lượng: tổng số có 24 đối tượng tham gia trong nhóm phỏng vấn trong đó có 11 nữ chiếm 46% và 13 nam chiếm 54%.
- Độ tuổi: đối tượng tham gia phỏng vấn ở độ tuổi từ 25 – 55 tuổi, trong đó ở độ tuổi từ 25 – 35 chiếm 25%, từ 35 – 45 chiếm 42 % và từ 45 -55 tuổi chiếm 33%.
- Nghề nghiệp: Kết quả phỏng vấn cho thấy, ngành nghề mà NKT làm rất đa dạng nhưng chủ yếu là lao động phổ thông. Số NKTcó việc làm chiếm 87% và không có việc làm chiếm 13%. Bảng số liệu dưới đây sẽ cho biết cụ thể về các loại nghề mà NKT đang làm.

**Bảng 1**: Nghề nghiệp của NKT

| **Nghề nghiệp** | **Số lượng** | **Tỷ lệ(%)** |
| --- | --- | --- |
| May | 7 | 33% |
| Mở công ty, xưởng sản xuất | 2 | 10% |
| Mở cửa hàng, kinh doanh tạp hóa | 3 | 14% |
| Làm việc ở tổ chức xã hội | 2 | 10% |
| Nông nghiệp | 2 | 10% |
| Ngành nghề khác | 5 | 24% |

Qua bảng số liệu, ta thấy nghề nghiệp chính của NKT tham gia phỏng vấn là nghề may chiếm 3329% và 100% là do nữ làm, tiếp theo là mở cửa hàng nhỏ kinh doanh tạp hóa chiếm 14% và cũng 100% do nữ làm, đứng thứ 3 đó là mở công ty, xưởng sản xuất, làm việc ở tổ chức xã hội và nông nghiệp chiếm 10%, các ngành nghề khác chiếm 24% như chạy xe ba bánh, cắt tóc, bán hàng vỉa hè, trồng cây cảnh…

- Trình độ học vấn: chủ yếu chưa tốt nghiệp phổ thông cấp 3 và chỉ có 4% tốt nghiệp đại học.

1. **Kết quả buổi phỏng vấn**
   1. **Khó khăn**

- **Khó khăn trong vấn đề xin việc:** Kết quả buổi phỏng vấn cho thấy tỷ lệ những người gặp khó khăn trong quá trình xin việc chỉ chiếm có 8% và họ đang ở độ tuổi 25-35. Nguyên nhân họ gặp khó khăn bởi (1) do họ là NKT, (2) do không có bằng cấp, chứng chỉ đào tạo nghề nghiệp cụ thể, (3) tiếp cận thông tin khó khăn, (4) ngại đến những cơ sở, những trung tâm giới thiệu việc làm và (5) không có sự hỗ trợ của gia đình.

Đối với 92% số người còn lại thì họ không phải không gặp khó khăn, vấn đề là 92% người này họ tự tạo việc làm cho chính mình bởi họ cho rằng 1 phần mình có đi xin việc thì nhà tuyển dụng cũng không tuyển bởi điều kiện sức khỏe của họ, một phần cũng do họ tự tạo ra được công việc phù hợp với tình hình sức khỏe và hoàn cảnh, điều kiện của họ. Họ chủ yếu làm các nghề phổ thông, thu nhập thấp như số liệu thống kê tại Bảng 1.

- **Khó khăn về tài chính:** 75% người trong nhóm phỏng vấn chia sẻ họ gặp khó khăn về tài chính. Do điều kiện sức khỏe bản thân cho nên đã ảnh hưởng tới khả năng tạo thu nhập và kinh tế của gia đình.

Trường hợp của anh T. bị khuyết tật ở cột sống, rất khó khăn trong việc cử động, không làm được việc gì, gia đình lại đông người, lại phải nuôi 3 con ăn học. Một mình vợ của anh phải bươn trải để nuôi sống gia đình. Thu nhập của gia đình anh chỉ là từ làm nông nghiệp, hết thời vụ, thì vợ anh lại phải đi tìm kiếm các việc làm thuê khác để có thêm thu nhập cho gia đình. Tuy nhiên, mức thu nhập cũng rất thấp và bập bõm từ việc làm thuê.

Một trường hợp khác nhà anh H. thì do công việc rất thất thường, lúc có lúc không. Gia đình anh có 4 người, 2 con nhỏ đang đi học. Do thu nhập không ổn định, chi phí học hành cho con cái lại rất tốn kém do đó gánh nặng về kinh tế luôn là vấn đề khó khăn nhất đối với gia đình anh.

Những người gặp khó khăn về tài chính thì chủ yếu là những người có sức khỏe yếu, gia đình đông con, một mình nuôi con, độc thân… Những nguyên nhân này khiến cho bản thân và gia đình của những NKT gặp khó khăn về tài chính. Sức khỏe yếu không làm được việc cộng với công việc lại không ổn định, thu nhập bấp bênh, thấp. Ngoài ra, sức khỏe yếu lại thường xuyên phải đi viện nên ảnh hưởng rất nhiều tới điều kiện kinh tế của gia đình.

- Kết quả phỏng vấn cũng chỉ ra rằng những người khuyết tật vận động này họ gặp **khó khăn trong việc đi lại,** đi đâu họ cũng phải có người trở hoặc phải đi xe ôm, taxi. Còn việc tiếp cận với các phương tiện giao thông công cộng như xe bus thì gần như họ không tiếp cận được bởi họ không thể tự mình lên xuống xe bus nếu như không có sự trợ giúp từ người khác.

Như một chia sẻ của bạn H trong nhóm phỏng vấn, bạn đưa ra một thực trạng nhu cầu sử dụng xe buýt của những bạn sinh viên người khuyết tật. Do điều kiện kinh tế khó khăn nên các bạn sinh viên cũng không có điều kiện để đi taxi mà đi xe bus thì không được do không thể tự mình lên xuống xe được.

Có người thì cũng muốn tự trang bị cho mình phương tiện đi lại cá nhân để chủ động trong việc đi lại và giảm chi phí thì tìm mua phương tiện lại rất khó. Chị T. chia sẻ chị rất muốn mua 1 chiếc xe đạp điện để đi lại cho thuận tiện và cũng đỡ mệt vì hiện tại chị đi lại dùng chiếc xe đạp cũ của mình. Tuy nhiên, do chị bị khuyết tật ở tay, cho nên gặp khó khăn trong việc phanh xe nên việc mua xe cũng không thực hiện được.

- **Khó khăn trong việc tiếp cận với cơ sở hạ tầng, các công trình công cộng** thì những người tham gia phỏng vấn cũng chia sẻ họ gặp rất khó khăn, như việc đi đến các nơi công cộng, cơ quan thì rất ít nơi có đường thiết kế riêng cho người khuyết tật, đặc biệt là khu vệ sinh. Chia sẻ về vấn đề này, một thành viên trong nhóm tham gia phỏng vấn đã đưa ra ngay một minh chứng rất thực tế và rõ ràng đó là ngay tại nơi diễn ra buổi phỏng vấn ngày hôm nay thì có chỗ xe lăn lên được, có chỗ lại không có đường cho xe lăn lên.
- **Thái độ của cộng đồng, gia đình đối với NKT:** Kết quả buổi phỏng vấn cho thấy trong xã hội vẫn còn có sự kỳ thị, thái độ đối với người khuyết tật. và không chỉ trong xã hội mà chính trong gia đình người khuyết tật cũng có sự kỳ thị đối với họ. Thái độ coi thường khả năng NKT, cho rằng họ vô dụng, không làm được việc gì. Một chia sẻ của trong buổi phỏng vấn về vấn đề này: *“…NKT nói chung chưa được xã hội nhìn nhận đúng mức họ vẫn coi thường NKT nhiều quá. NKT thường hay mặc cảm với xã hội vì họ cảm thấy người bình thường coi thường họ, cảm thấy kém cỏi so với người bình thường mặc dù trên thực tế họ không thua kém người bình thường, thậm chí có những việc mà NKT làm được mà người bình thường không làm được. Vì NKT tuy bị thiếu hụt hơn người lành lặn nhưng NKT vươn lên bằng nghị lực, trí não nên có những việc họ làm được mà người bình thường không làm được. Nhưng người bình thường hay nhìn nhận chắc là NKT khó khăn lắm không làm được như người BT. Trước kia tôi cũng nghĩ rằng không biết có làm được những việc như thế không nhưng sau này tôi cũng thích nghi dần dần và có thể tự lực làm được mọi việc. VD như tôi có thể bưng 1 bát cháo to lên cho mẹ tôi bằng cách cho vào thùng nhỏ đeo bên hông và đi bằng nạng….”*. Một chia sẻ khác của chị L trong buổi phỏng vấn *“…mọi người nhìn thấy tôi luôn hỏi tôi có thể làm được gì không tôi trả lời bạn làm được gì tôi cũng có thể làm được. Bạn làm theo cách của bạn tôi làm theo cách của tôi”.* Qua đây cho thấy rằng, bản thân người khuyết tật cũng mặc cảm và tự ti về chính mình điều này cũng một phần do xã hội mang lại, tuy nhiên sự mặc cảm, tự ti này chính bản thân họ dần dần cũng tìm cách khắc phục, vượt qua và chứng minh bằng chính sự nỗ lực, sự vươn lên của bản thân từ việc đơn giản như tự chăm sóc bản thân đến việc tự tạo ra việc làm phù hợp với tình trạng khuyết tật và khả năng của mình.
- **Việc tiếp cận các nguồn thông tin:** Việc tiếp cận các nguồn thông tin như các chính sách trợ cấp, các chế độ cho người khuyết tật nói chung ở mức độ vừa phải. Chưa có nhiều các hoạt động, cách thức để tuyên truyền, giới thiệu về các chương trình, chính sách hỗ trợ cho người khuyết tật. Những thông tin mà NKT có được chủ yếu là thông qua các cuộc họp của Hội NKT tại địa phương, thông qua truyền tai nhau người này bảo người kia. Chú T. chia sẻ: chú *có được thông tin là bởi con của chú làm ở Thông tấn xã Việt Nam, cho nên chú cũng hay hỏi và biết đến những văn bản, chính sách liên quan đến người khuyết tật.*

Trong số những người tham gia phỏng vấn thì cũng có trường hợp chia sẻ là họ hầu như không có thông tin gì cả.

- 1. ***Chi phí phát sinh***

Buổi phỏng vấn cho thấy, trong cuộc sống hàng ngày của NKT có rất nhiều các chi phí phát sinh. Tuy nhiên, tập trung nhiều và phổ biến là những chi phí phát sinh sau:

- **Các chi phí y tế:** Do đặc điểm bản thân bị khuyết tật cho nên tình trạng sức khoẻ của những NKT cũng bị ảnh hưởng ít nhiều. Vì vậy, việc hay phải đi thăm khám tại các cơ sở y tế là rất thường xuyên với họ. Đối với những người có thẻ BHYT thì chi phí phát sinh đó là tiền khám thêm, mua thêm thuốc bên ngoài bởi trong thẻ bảo hiểm y tế thì họ chỉ được hưởng trợ cấp một số loại thuốc còn những loại thuốc khác thì họ phải tự bỏ tiền túi ra để mua. *Có trường hợp trong nhóm phỏng vấn bị bệnh ngừng thở khi ngủ, điều trị bệnh này thì lại không nằm trong diện bảo hiểm y tế, các thiết bị hỗ trợ cho việc điều trị này cũng vậy. Do vậy, họ phải chi rất nhiều tiền cho việc điều trị.*

Còn đối với những người không có thẻ BHYT thì họ phải chi trả như một người bình thường. *Chia sẻ của chị T. trong buổi phỏng vấn về trường hợp của mình: Chị không có thẻ bảo hiểm y tế, lại bị mắc bệnh thiên đầu thống. Chi phí để chị phải chi trả cho mỗi lần đi mổ là rất tốn kém và chị phải chi trả toàn bộ. Chị đã phải đi mổ đến lần thứ 4 và đã 2 năm nay chị chưa đi khám lại từ sau lần mổ cuối bởi chị sợ khi khám lại có thể chị sẽ lại phải yêu cầu mổ mà chị thì chưa có tiền.*

- Ngoài chi phí phải bỏ thêm tiền để mua thuốc ngoài bảo hiểm và trong quá trình điều trị thì những chi phí khác đi kèm khi NKT đi khám chữa bệnh đó là **chi phí cho người thân trong gia đình đi theo để giúp họ trong việc khám chữa bệnh, điều trị** (đi lại tăng thêm, ăn ở nếu phải nằm viện…)
- **Chi phí đi lại:** do là người khuyết tật vận động nên họ phải bỏ ra nhiều các chi phí cho việc đi lại bởi họ gặp rất khó khăn trong việc di chuyển: họ phải thuê xe ôm hay đi taxi để di chuyển.
- **Chi thiết bị hỗ trợ:** Để có thể tự mình di chuyển, thì cũng có một số những NKT họ chủ động trang bị các phương tiện di chuyển phù hợp với mình như xe 3 bánh, nạng… và với họ thì đây đều là các chi phí phát sinh. Bởi ví dụ khi họ mua 1 chiếc xe máy bình thường về nếu với người bình thường thì chỉ việc sử dụng luôn nhưng đối với họ thì lại phải sửa chữa, thay đổi một số chi tiết và thiết kế để họ có thể sử dụng được. Và để thực hiện những điều này thì đều phát sinh các chi phí
  1. ***Trang trải các chi phí phát sinh***

Để trang trải các khoản chi phí phát sinh và trong cuộc sống hàng ngày thì kết quả buổi phỏng vấn cho thấy một số hướng giải quyết sau:

- **Vay vốn tín dụng:** vay vốn tín dụng để mở rộng hoạt động sản xuất kinh; để xây nhà trọ cho thuê, mở cửa hàng … tạo thêm thu nhập cho gia đình;
- **Vay mượn của người thân và bạn bè:** để đóng học cho con; để đi chữa bệnh.
- **Đi tìm các việc thời vụ để có thu nhập trang trải các chi phí.**
  1. ***Hỗ trợ của người thân trong gia đình***

Qua phỏng vấn cho thấy đa số tất cả những NKT tham gia phỏng vấn đều nhận được sự hỗ trợ của người thân. Những sự hỗ trợ này thì dưới nhiều hình thức như hỗ trợ chăm sóc cho bản thân những người khuyết tật trong cuộc sống hàng ngày, có người thì nhận được sự hỗ trợ tiền bạc của những người thân, họ hàng, có người thì nhận được sự hỗ trợ của người thân thông qua việc người thân tạo môi trường và luôn tạo cơ hội để bản thân NKT đó cảm thấy mình luôn hòa nhập với cộng đồng và có thể tự mình chăm sóc và nuôi dưỡng bản thân, tự tin vào khả năng của mình.

Tuy nhiên, cũng có người không nhận được sự hỗ trợ nào của người thân trong gia đình, họ phải tự mình lo hết mọi thứ từ việc chăm sóc bản thân cho đến việc tìm kế sinh nhai.

- 1. ***Hỗ trợ từ phía Nhà nước và cộng đồng***

Kết qủa phỏng vấn cho thấy NKT nhận được các sự trợ giúp từ phía Nhà nước và cộng đồng xã hội. Cụ thể, NKT nhận được những sự trợ giúp thông qua các chính sách sau:

- **Chính sách trợ cấp cho NKT:** Cuộc phỏng vấn cho thấy không phải tất cả NKT đều được nhận trợ cấp xã hội, có 67% NKT được nhận trợ cấp xã hội và 33% không được nhận trợ cấp. NKT đánh giá cao chính sách trợ cấp của Nhà nước tuy nhiên họ cũng đề nghị là được nâng mức trợ cấp lên vì mức trợ cấp hiện tại tương đối thấp.
- **Chăm sóc sức khỏe:** Kết quả phỏng vấn cho thấy có 67% người có thẻ BHYT và và 33% không có thẻ BHYT. Như vậy, không phải NKT nào cũng được hưởng chế độ từ BHYT.
- **Chính sách vay vốn:** 100% những người tham gia phỏng vấn đều được vay vốn với hạn mức tối đa là 20.000.000 đồng với thời hạn là 2 năm và mức lãi suất là 0,03%/tháng. Thủ tục vay đơn giản, dưới 20.000.000đ không cần thế chấp, trên 20.000.000đ xuất trình giấy đăng ký kinh doanh. Lãi suất 1 tháng hoặc 3 tháng trả 1 lần. Họ đánh giá chính sách này rất thiết thực, hiệu quả phù hợp với nhu cầu mong muốn và khả năng chi trả của họ bởi họ chỉ phải chịu mức lãi suất rất thấp và thời gian vay cũng tương đối dài.
- **Các chính sách miễn giảm:**

Qua buổi phỏng vấn, chính sách miễn giảm của Nhà nước như đóng tiền đèn đường, vệ sinh thì có 1 người trong nhóm chia sẻ họ được hưởng chính sách này.

Chính sách miễn giảm cho NKT khi đi học: NKT đều được hưởng.

Chính sách miễn giảm cho con NKT: Qua phỏng vấn cho thấy đa số con em của NKT không nhận được bất kỳ miễn giảm nào (như giảm học phí, khi sử dụng các phương tiện công cộng…) tuy nhiên cũng qua chia sẻ của 1 người trong nhóm phỏng vấn thì con em NKT được hưởng những chế độ miễn giảm khi đi học.

- **Vé xe bus miễn phí:** Đa số NKT không sử dụng vé xe bus miễn phí bởi vì họ không sử dụng được. Việc tiếp cận sử dụng xe bus quá khó khăn đối với họ và đặc biệt có một vấn đề đó là vé xe bus ở vùng nào thì chỉ có giá trị ở vùng đó. *Một NKT trong buổi phỏng vấn chia sẻ: khi tôi đi ra tỉnh ngoài, đưa vé xe bus miễn phí ra thì anh phụ lái nói luôn rằng anh mang cái vé này về Hà Nội mà dùng, chúng tôi ở đây không dùng.* Một vấn đề khác đó là việc linh hoạt khi sử dụng vé xe bus. Ví dụ, như khi đi xe bus ở Hà Nội, nếu như NKT không đưa vé xe bus cấp cho NKT ra thì họ vẫn thu tiền như của người bình thường. Trong khi đó, ở Thành phố HCM, NKT không cần đưa vé xe bus miễn phí ra thì họ vẫn được miễn phí khi lên xe bus.
  1. **Loại hình giúp đỡ từ Chính phủ hay người thân trong gia đình:**

Kết quả phỏng vấn cho thấy NKT mong muốn nhận được sự hỗ trợ của Nhà nước trên những vấn đề sau:

- Chính sách chăm sóc sức khỏe: mong muốn Nhà nước cấp thẻ BHYT miễn phí cho tất cả NKT vì trên thực tế không phải NKT nào cũng được cấp thẻ BHYT, chỉ những người nào được nhận trợ cấp xã hội thì mới được nhận thẻ BHYT.
- Chính sách bảo trợ xã hội: Mong muốn mọi NKT đều được nhận trợ cấp xã hội bởi trên thực tế không phải NKT nào cũng được nhận trợ cấp xã hội để giảm bớt khó khăn cho NKT. Đồng thời, cũng mong muốn nâng mức trợ cấp xã hội lên.
- Các chính sách miễn giảm khác: mong muốn có chính sách miễn giảm các loại thuế giá trị gia tăng cho NKT. Ví dụ, tiền điện, nước, internet…
- Chính sách miễn giảm giá vé, giá dịch vụ khi tham gia sử dụng các dịch vụ phương tiện công cộng như tàu hỏa, máy bay; khi sử dụng dịch vụ văn hóa, thể thao, giải trí và du lịch tại các cơ sở văn hóa, thể thao: mong muốn được giảm một phần giá khi sử dụng các dịch vụ này để tạo điều kiện cho NKT hòa nhập với cộng đồng.
- Chính sách miễn giảm cho con em NKT: Qua phỏng vấn, hầu như con em NKT không được hưởng bất kỳ sự hỗ trợ hay miễn giảm nào, như học phí và các khoản đóng góp thêm ở nhà trường, tham gia các phương tiện giao thông công cộng. Gia đình họ vẫn phải đóng các khoản phí giống như con em của các gia đình bình thường. Do vậy, họ mong muốn có chính sách hỗ trợ của Nhà nước cho con em của họ.
- Công việc: Qua phỏng vấn, có một số NKT họ mong muốn có được công ăn việc làm ổn định để có thể nuôi sống bản thân, duy trì cuộc sống gia đình và giảm bớt gánh nặng cho gia đình.

**Báo cáo Nhóm Khiếm Thính 18/09**

**Về Bảo trợ Xã hội và Chính sách**

**Với Người Khuyết tật Việt Nam**

Trong khuôn khổ dự án Bảo trợ Xã hội và Chính sách, chúng tôi đã tiến hành thảo luận nhóm với một nhóm người khiếm thính vào buổi sáng thứ 4 ngày 18 tháng 9 năm 2013 tại trường Estih, số 1, ngõ 75, phố Đặng Văn Ngữ, quận Đống Đa, Hà Nội.

Nhóm khiếm thính được phỏng vấn ngày 18/09 gồm 9 người, trong đó có 5 nam và 3 nữ khiếm thính, và 1 nữ phiên dịch viên. Tổng thời gian làm việc là 2 tiếng đồng hồ tính từ thời điểm tập hợp nhóm lúc 9h00, bắt đầu thảo luận lúc 9h30, và kết thúc vào lúc 11h10.

Hình thức thảo luận nhóm là người phỏng vấn đưa ra 6 câu hỏi lớn và khuyến khích các thành viên trong nhóm khiếm thính tự chia sẻ các thông tin liên quan. Trong suốt buổi thảo luận, các thành viên đã nhiệt tình chia sẻ quan điểm cá nhân, các vấn đề được đề cập, cũng như nêu lên một số mong muốn của bản thân.

Độ tuổi trung bình của nhóm là 19 – 23 với 4 thành viên hiện đang theo học một chương trình giáo dục tài trợ bởi Chính phủ Nhật. Các thành viên còn lại có 2 người ở độ tuổi trên 30 và 2 người dưới 23 tuổi. Trong số 4 người này, có 3 người lớn tuổi nhất đã lập gia đình và có việc làm còn một thành viên còn lại khoảng 21 tuổi hiện đang thất nghiệp.

Với câu hỏi thứ nhất, khi được hỏi về ***các vấn đề khó khăn trong quá trình xin việc làm (câu 1)***, một thành viên đã đại diện nhóm tóm lược một số khó khăn cơ bản mà đa số cả nhóm đều gặp phải. Đầu tiên là ***sự phân biệt đối xử của nhà tuyển dụng*** *(c)* cũng như là ***trình độ văn hóa*** *(d)* của bản thân những người khiếm thính chưa đáp ứng được yêu cầu: *"Bản thân tôi là một người khiếm thính, và bản thân kinh nghiệm của tôi, thì tôi thấy là cái việc xin việc làm có rất nhiều rào cản. Bởi vì bản thân chúng tôi chưa có nhiều kinh nghiệm, bởi vậy mà khi xin việc làm chúng tôi gặp rất nhiều rào cản."* (T.A.) (6.57)

Khi nói về vấn đề ***phân biệt đối xử*** *(c)*, ghi nhận từ phía những người khuyết tật cho thấy nhiều nhà tuyển dụng nhìn nhận người khiếm thính chỉ đơn thuần là những người khuyết tật nên khó đáp ứng được yêu cầu công việc nói chung. Nhưng thực tế những người khiếm thính cho rằng họ chỉ có vấn đề về việc nghe - nói trong giao tiếp còn về cơ bản các việc cần nhiều về sức khỏe cũng như các công việc trí óc họ dều có thể đáp ứng được. Điển hình trong nhóm có một thành viên tên K., 31 tuổi, đang làm cho một công ty tin học là một ví dụ minh chứng cho khả năng làm việc của người khuyết tật nói chung, người khiếm thính nói riêng.

Tuy vậy, không phải thành viên nào cũng may mắn như vậy, với bản thân anh T. thì chia sẻ rằng: *"Hồi trước tôi có làm cái hồ sơ để nộp vào cái khách sạn Nicko. Và sau đó là tôi để hồ sơ đó, ở đó rất là lâu, và tôi nghĩ là họ có thể cần thời gian để kiểm tra năng lực."* (9.51) Hồ sơ của một số thành viên thường không được các nhà tuyển dụng quan tâm, họ hứa hẹn xem xét và chỉ đơn thuần là không liên lạc lại. 2 thành viên có việc làm của nhóm còn lại, 1 là có sự trợ giúp của ngôi trường anh đã từng theo học để làm công tác đào tạo chuyên viên về người khiếm thính còn 1 là mở một quán nước với thu nhập không ổn định.

Rõ ràng, vấn đề phân biệt đối xử chính là rào cản lớn nhất để người khiếm thính bước chân vào một công ty thực sự ngoài thị trường.

Về ***trình độ văn hóa*** *(d)*, người khiếm thính gặp phải một rào cản lớn là bậc học của họ, đặc biệt là với những người khiếm thính ở ngoài Hà Nội, chỉ đạt đến cấp 2 và thường họ chỉ học xong cấp 1. Trong Sài Gòn thì các bạn khiếm thính có trình độ văn hóa cao hơn, và có thể học đến đại học. Anh T.A. chia sẻ: *"Tôi xin chia sẻ một chút là cái thông tin về cộng đồng người điếc ở Việt Nam, thì đó là ở Miền Nam các bạn ấy có được học lên cái trình độ cấp 1, cấp 2, cấp 3, và thậm chí là đại học nhưng mà ở Hà Nội thì hiện tại chỉ có đến trình độ cấp 2 thôi. Và các bạn ở miền Nam thì có chia sẻ là trong đó thì các bạn học xong rồi nhưng mà vẫn khó khăn trong việc xin việc làm."* (17.17) Với yêu cầu khắc nghiệt của thị trường việc làm hiện nay, việc chưa hoàn thành 12 năm phổ thông là một vấn đề rất đáng lưu tâm với người khiếm thính.

Bên cạnh đó, vấn đề ***tìm kiếm thông tin việc làm*** *(h)* cũng là một trở ngại cho người khiếm thính. Họ phải nhờ bạn bè trong cộng đồng người khiếm thính nói riêng và người khuyết tật nói chung để có được những thông tin về các khóa học cũng như cơ hội việc làm.

Người khiếm thính thường gặp phải vấn đề về ***giao tiếp với mọi người*** *(i)* trong xã hội bên ngoài. Chị H. chia sẻ: *"Đó là việc giao tiếp giữa người khiếm thính và người nghe nói. Và tôi cảm thấy là cái rào cản đó là khi mà, khi mà người khiếm thính họ chỉ hòa đồng trong cái thế giới của họ thôi. Và người nghe nói cũng vậy, khi mà giao tiếp với nhau thì trở ngại và một phần, một phần cũng là do cái ngôn ngữ khác nhau cho nên là rất ngại để hòa đồng với nhau. Và người nghe nói thì luôn nghĩ là cái ngôn ngữ của , tức là ngôn ngữ kí hiệu chỉ là một cái điệu bộ. Không phải là một ngôn ngữ. Chỉ là những cái hành động hài hước, gây cười cho người ta thôi. Nhưng mà đấy thì không phải, tôi nghĩ đấy là sai."* (8.54) Vì khuyết tật của mình, người khiếm thính phải sử dụng điện thoại di động hay giấy viết để giao tiếp với người bình thường, không phải ai cũng thấy thoải mái để trao đổi lại, một số người thậm chí đã tỏ thái độ khó chịu. Người khuyết tật thường nhạy cảm, vậy nên việc giao tiếp càng trở nên khó khăn.

***Thái độ của gia đình*** *(b)* cũng đóng một vai trò lớn. Một số thành viên đã được sự ủng hộ tích cực của gia đình tuy nhiên với một số gia đình khác, thì họ đã tỏ lo lắng về khả năng học lên cao để xin việc của các thành viên này. Như chị N.A. chia sẻ: *"Tôi nghĩ rằng bố mẹ tôi lo lắng tương lai tôi đi học xong thì tôi sẽ làm cái gì và tôi đóng góp cho xã hội như thế nào? Bố mẹ tôi lo rất là nhiều tại vì là lo nếu mà người bình thường, người nghe nói không lo nhưng mà bản thân tôi bị điếc, cái gánh nặng đó sẽ nhiều hơn."* (20.01) Thái độ của gia đình sẽ là động lực ban đầu để người khiếm thính bước chân ra ngoài xã hội và cũng sẽ là viên đá cản đường đầu tiên nếu những phản hồi là không tích cực.

Khi đề cập đến ***những khoản chi phí phát sinh*** *(câu 2)* của bản thân người khiếm thính, một số thành viên chia sẻ rằng họ rất khó trang trải những chi phí y tế nói chung cũng như không đủ khả năng để mua ***thiết bị hỗ trợ*** *(a)*. Máy trợ thính là dụng cụ hỗ trợ cần thiết và toàn bộ các thành viên đều chỉ cần mua máy này. Tuy nhiên, vấn đề giá cả đã khiến họ không tiếp cận được các máy móc hiện đại và việc giao tiếp nhìn chung chỉ dựa trên ngôn ngữ kí hiệu trong cộng đồng người khiếm thính hoặc qua điện thoại hay giấy bút với người nghe nói.

Với đặc thù khuyết tật của mình, người khiếm thính không gặp nhiều khó khăn về việc di chuyển hay vấn đề cơ sở vật chất không đáp ứng được nhu cầu của họ. Các chi phí y tế khác, nếu là liên quan tới vấn đề khiếm thính, cũng không được các thành viên đề cập. Còn bảo hiểm y tế, về cơ bản là không có phần hỗ trợ riêng cho người khiếm thính nên các thành viên phải tự mua hoặc được hỗ trợ nếu là đang theo học trong trường. Nếu đã rời khỏi trường học, người khiếm thính sẽ phải mua bảo hiểm y tế với giá cả tương đương với người bình thường. Chưa kể khi đi khám, rất khó để bác sĩ hỏi các triệu chứng bệnh vì họ phải trao đổi qua giấy hay điện thoại nên nhiều khi việc khám bệnh rất khó khăn hoặc không có sự hợp tác.

Để ***trang trải những khoản chi phí trên*** *(câu 3)* cũng như các chi phí sinh hoạt đời thường khác, ngoài số tiền kiếm được từ công việc của họ, các thành viên trong nhóm vẫn phải vay mượn thêm từ bạn bè, gia đình, và họ hàng. Những khoản vay này thường không quá nhiều, trong khoảng từ 2-3 triệu cho một gia đình như của T.A. chia sẻ. Hoặc có thể là một số tiền ít hơn để có thể trả ngay trong một khoảng thời gian ngắn. Về cơ bản, các thành viên phải tự cắt giảm chi tiêu với những nguồn thu nhập ít ỏi, như H. (21 tuổi) chia sẻ rằng em luôn tránh phải phụ thuộc tài chính vào bố mẹ.

Một số thành viên đang đi học đã tự trang trải bằng cách làm thêm như mở quán trà chanh hay bán quần áo tại nhà. Tuy vậy, những công việc này chỉ mang tính tạm thời vì thu nhập hạn chế và cũng vì các thành viên này vẫn đang theo học tại trường.

Tuy trong cuộc sống, tài chính của các thành viên rất thiếu thốn và họ thường xuyên phải tìm kiếm sự giúp đỡ nhưng những nguồn cho vay thì lại rất hạn chế. Bạn bè và người thân luôn là hai nhóm chính và có lẽ là duy nhất mà các thành viên có thể tìm đến khi cần thiết. Không chỉ về mặt tài chính, mà đôi khi ***sự giúp đỡ của gia đình, người thân*** *(câu 4)* về vấn đề giao tiếp hàng ngày như khi đi khám bệnh cũng như là một sự hỗ trợ đáng kể.

Những việc mà gia đình, người thân hỗ trợ các thành viên trong nhóm là rất nhiều, có thể chia làm 3 dạng cơ bản là động viên tinh thần, giúp đỡ tài chính, và hỗ trợ việc làm. Sự giúp đỡ thứ 3 là sự hỗ trợ rất lớn với các thành viên vì giai đoạn tìm được một công việc ổn định mất rất nhiều thời gian, chưa kể 4 thành viên đang đi học tới đây sẽ phải tự bươn chải sau khi ra trường. Bản thân người khiếm thính luôn nỗ lực để tìm được một việc làm có thể nuôi sống họ, nhưng nếu không có sự trợ giúp từ người thân, gánh nặng tinh thần sẽ là không thể tránh khỏi.

Tuy vậy, sự hỗ trợ của gia đình chỉ mang tính tạm thời, nhiều chi phí về y tế và giáo dục cũng như các chính sách về việc làm cho người khiếm thính lại chưa nhận được sự quan tâm xác đáng của Nhà nước. Theo anh T.A., những ***sự hỗ trợ*** *(câu 5)* này sẽ có ý nghĩa rất lớn về lâu dài và làm giảm gánh nặng kinh tế cho gia đình của những người khuyết tật.

Những thành viên trong nhóm cũng đưa ra ***một số mong muốn*** *(câu 6)* chủ yếu như sự đãi ngộ của Nhà nước về việc ***chăm sóc sức khỏe và khả năng phục hồi chức năng*** *(c)* dành riêng cho người khiếm thính, ví dụ như có người phiên dịch ngôn ngữ kí hiệu trong các bệnh viện lớn. ***Những khoản trợ cấp hàng tháng*** *(b)* để họ có thể trang trải cuộc sống hàng ngày cũng là một đòi hỏi thiết yếu.

Ngoài ra, các thành viên trong nhóm cũng mong muốn sự thay đổi trong ***nhận thức của các doanh nghiệp cũng như toàn xã hội*** *(e)* để nhận người khuyết tật vào làm việc và có đãi ngộ công bằng như những người bình thường khác cũng như để họ có thể thật sự hòa nhập vào cộng đồng. Họ cũng mong rằng trong các chương trình truyền hình sẽ có nhiều chương trình có các ngôn ngữ kí hiệu để phục vụ cho nhu cầu giải trí của họ.

Cuối cùng, về vấn đề ***giáo dục*** *(d)*, người khiếm thính mong rằng họ sẽ được học hành lên cao để có được những cơ hội việc làm ổn định hơn.

**BÁO CÁO KẾT QUẢ PHỎNG VẤN**

Về “Bảo trợ xã hội và chính sách với người khuyết tật Việt Nam” được tiến hành tại:

- Địa điểm: Trường Trung Cấp Tin Học Hà Nội (Số 1 – Ngõ 75 – Đặng Văn Ngữ - HN)
- Thời gian: 9g00 – 11g00, thứ Tư ngày 18 tháng 9 năm 2013.

Thông tin chung về nhóm đối tượng tham gia trả lời phỏng vấn.

- Khuyết tật vận động: 01
- Khiếm thị: 04
- Khuyết tật nói: 01
- Chậm phát triển: 01
- Phụ huynh có con em bị bại não, tự kỷ, chậm phát triển: 04

Số lượng: Có 11 người (6 nữ, 5 nam)

Độ tuổi của nhóm: từ 25 – 62

Nghề nghiệp; Quản lý, nhân viên cơ quan, nội trợ, bấm huyệt, tự doanh, chưa có việc làm.

Trình độ học vấn: đại học có 1 người khiếm thị, 1 chậm phát triển, 2 phụ huynh. Còn lại là chưa tốt nghiệp phổ thông.

**Kết quả buổi phỏng vấn:**

Nội dung buổi phỏng vấn tập trung trả lời 6 câu hỏi sau:

**Câu hỏi 1: Những người khuyết tật thường gặp nhiều khó khăn trong vấn đề xin việc làm. Anh (chị) đã từng gặp phải những khó khăn đó như thế nào?**

**1/. Khó khăn về việc làm:** Kết quả buổi phỏng vấn cho thấy chỉ có 3/11 người có nhu cầu tìm việc và họ đang ở tuổi 25 và 35. 3 người này chia sẻ họ gặp rất nhiều khó khăn trong việc xin việc

- Khó khăn về khả năng tìm kiếm thông tin việc làm. Do bị khiếm thị và chậm phát triển nên 1 số thành viên khó khăn khi sử dụng được máy vi tính, nên khả năng tìm hiểu thông tin việc làm còn gặp nhiều hạn chế.
- Khó khăn về trình độ học vấn. Có 2/11 người đã tốt nghiệp đại học còn các thành viên khác đều ở trình độ PTTH. Chỉ có 2/11 thành viên có khả năng sử dụng thành thạo kỹ năng tin học và phần lớn trong số họ là lao động thủ công như ( xoa bóp bấm huyệt, tự doanh tại nhà…)
- Khó khăn về khả năng đi lại. Những thành viên khiếm thị và khuyết tật vận động gặp khó khăn rất lớn trong việc di chuyển. Những thành viên khiếm thị như chị L, bác T, và bác M khó xác định phương hướng, việc đi lại thường phải phụ thuộc vào người khác. Thành viên khuyết tật vận động như bác D cũng gặp khó khăn về phương tiện như: tự đi xe máy hay xe đạp - phương tiện chủ yếu là xe bus, nhưng thái độ phục vụ của lái xe thì không tốt. Bác D nói: *“mỗi lần đi xe buýt thường bị rớt lại, có những lần phải chờ 2- 3 tiếng vẫn chưa lên được xe”*
- Khó khăn là sự phân biệt đối xử của các nhà tuyển dụng. Như trường hợp của chị M, nhiều lần chị đã nộp hồ sơ xin việc trên diễn đàn, khi được mời đến phỏng vấn thì nhà tuyển dụng lại trả lời là đã nhậnđược người rồi“có lẽ, khi họ nhìn thấy tôi khó khăn khi giao tiếp và sức khỏe yếu, nên họ nói vậy” . Hay trường hợp của anh G, bị ngã khi đang học cấp 2, anh bị ảnh hưởng não nên khả ngăng tiếp thuvà nhận thức chậm hơn bình thường. Hiện anh đã tôt nghiệp đại học giao thông và trường trung cấp điện nhưng khi đi xin việc, nhà tuyển dụng phỏng vấn, thì anh thườngtrả lờikhá chậm với các câu hỏi ( do phải có thời gian dài để hiểu câu hỏi) đã không kiên nhẫn chờ đợi và không tạo điều kiện để nhận anh vào làm.
- Trở ngại nữa là sức khỏe hạn chế. Các thành viên thường có sức khỏe yếu nên không thể đảm nhận những công việc diễn ra trong thời gian dài hay có áp lực cao. Do vậy họ không thể làm việc trong môi trường cạnh tranh cao.
- Cuối cùng là khó khăn về tài chính. Tiêu biểu là chị M cho biết “tôi chỉ gặp khó khăn trong việc nói nhưng trí óc tôi vẫn hoạt động bình thường, có những việc làm được thì thu nhập lại quá thấp, không đảm bảo nhu cầu kinh tế”. Các thành viên chủ yếu làm công việc đơn giản như: nghề thủ công, mát xa, bấm huyệt nên thu nhập thấp, không ổn định.

**Câu 2: Trong gia đình của những người khuyết tật thường có những khoản chi phí phát sinh. Với cá nhân bạn, những chi phí đó là gì?**

**Khó khăn về tài chính:** 10/11 người trong nhóm chia sẻ họ gặp khó khăn về tài chính.

- Nhìn chung, khoản chi phí phát sinh mà hầu hết các thành viên trong nhóm đều thấy chiếm khá cao là chi phí y tế. Các thành viên đã chia sẻ chuyện của mình như: chị H (mẹ của cháu B) cho biết, con của chị đang phải điều trị bằng châm cứu, chi phí khoảng 10 triệu nhưng bảo hiểm y tế chỉ hỗ trợ 30%, 70% khoản chi phí đó vẫn là khá lớn với gia đình chị. Hay bác Đ, bác phải chịu nhiều khoản chi phí chữa trị mắt, u phổi, và tuyến giáp nhưng thu nhập từ công việc mat-xa, bấm huyệt của bác không thể đủ chi trả cho các khoản này. Hầu hết các thành viên đều có thẻ bảo hiểm y tế nhưng thẻ đó không đủ để chi trả các khoản chữa trị những căn bệnh của họ.
- Ngoài ra còn có thêm chi phí di chuyển. Mặc dù có được hỗ trợ thẻ miễn phí đi xe bus, nhưng phần lớn người khuyết tật vận động không thể đi xe bus nên họ phải đi xe ôm hoặc taxi nên chi cho việc này khá tốn kém. Như bác D, do mới đây bị tai nạn, chân bác bị ảnh hưởng nặng thêm nên thường đi lại bằng xe ôm mà khoản chi phí xe ôm cũng khá cao, vào khoảng 10.000 đồng/ km.
- Khoản chi phí nữa là giáo dục. Các con bị khuyết tật, nhất là bị tự kỷ hay chậm trí tuệ không được nhận vào các trường lớp chung với mức học phí bình thường mà phải vào các lớp chuyên biệt hoặc thuê người dạy ở nhà với mức phí cao hơn rất nhiều, khoảng từ 4-5 triệu đồng/tháng. Đó là trường hợp của con chị H và cháu của bác M.

**Câu 3: Làm thế nào anh/ chị trang trải được những chi phí này?**

- Hầu hết các thành viên trong nhóm không nhận được hỗ trợ từ phía nhà nước để trang trải những khoản chi phí phát sinh. Mọi người đều phải rất tiết kiệm để tự trả các chi phí này bằng chính thu nhập cá nhân
- Ngoài ra họ cũng nhận được sự hỗ trợ từ phía bố mẹ, anh em người thân trong gia đình, nhưng không đáng kể.
- Đôi khi các thành viên cũng nhận được hỗ trợ đi lại từ hàng xóm. Thi thoảng bác D cũng được hàng xóm cho đi nhờ và thăm hỏi động viên.
- Như trường hợp của chị H thường phải xin cơ quan cho tạm ứng lương tháng trước để có tiền chi phí cho con. Gần đây chị phải xin tạm ứng trước lương 3 tháng, nhưng cho đến giờ vẫn chưa trả được.

**Câu 4: Các thành viên khác đã hỗ trợ anh/ chị như thế nào?**

- Phần lớn mọi người đều nhận được sự hỗ trợ rất lớn từ phía gia đình như thời gian và tài chính:

**+ Chăm sóc:**

Hàng ngày các bậc phụ huynh phải dành một lượng thời gian khá lớn để chăm sóc con em mình. Như chị H, những hôm đưa con đi châm cứu thường phải xin đi làm muộn khoảng 2 tiếng, sau đó chị vẫn phải hoàn thành đủ 8 tiếng làm việc nên phải làm thông trưa hoặc làm tăng ca. Khi xin nghỉ 2 tiếng để đưa con đi châm cứu cũng rất khó khăn, nhiều khi chị không dám xin nghỉ nhiều vì sợ sẽ dễ bị đuổi việc. Hay như bác M đã phải nghỉ hưu sớm để ở nhà chăm người con trai 30 tuổi bị bại não và một người cháu 5 tuổi bị tăng động và tự kỷ. Còn cô C, về hưu rồi nhưng không đi dạy học thêm được dù thu nhập hàng tháng rất thấp chỉ khoảng 3 triệu đồng/tháng, vì phải dành toàn bộ thời gian để chăm sóc con, đưa con đi học và đi khám bệnh.

**+ Tài chính:**

Các chi phí như y tế, giáo dục, đi lại của các thành viên khuyết tật chỉ có gia đình hỗ trợ chi trả.

- Các thành viên khác trong gia đình không chỉ bị ảnh hưởng đến quỹ thời gian, làm việc và tài chính mà còn cả quan hệ xã hội. Nhiều khi đi ra ngoài thường nhận cái nhìn đố kị, phân biệt, xa cách của mọi người với tình trạng khuyết tật của con em mình. Thậm chí có người còn không cho con họ chơi cùng con của các chị, như chị H nói: “có lần chị đem cho bánh thì phải chờ chị và con chị đi về mới dám ăn”. Hay những định kiến là “đời cha ăn mặn đời con khát nước”, không biết chị làm gì mà cả con cả cháu đều không bình thường.

**Câu 5: Những khoản hỗ trợ nào mà các anh/ chị đã nhận được từ nhà nước hay từ các thành viên khác trong gia đình? Anh/ chị đánh giá thế nào về những sự hỗ trợ đó?**

- **Y tế:**

+ Đa số các thành viên khuyết tật được phát thẻ bảo hiểm y tế. Tuy nhiên, chi phí hỗ trợ từ bảo hiểm y tế đó không đáng kể nên hầu như ít khi dùng đến.

+ Việc giám định y khoa còn hời hợt, khám tập thể và mang tính chất rà soát. Đôi khi có hiện tượng tiêu cực, như khi muốn giám định kỹ lưỡng, hoặc đánh giá mức độ nặng – nhẹ thì lại phải có “phong bì” cho cán bộ y tế khám giám định.

+ Thủ tục khám chữa bệnh còn rườm rà và chưa có chế độ ưu tiên đúng mức cho người khuyết tật.

- **Giáo dục:**

**+** Khá nhiều người khuyết tật đi học nghề, học trung cấp hay đại học đều được giảm học phí hoặc được miễn toàn phần. Trong nhóm có chị M đi học trung cấp tin học được miễn phí.

+ Tuy nhiên, không phải ai cũng có được may mắn đó. Những trẻ em khuyết tật ở độ tuổi đi học như con của cô C, con anh T, con chị H, và cháu bác M còn gặp nhiều khó khăn khi xin cho con vào học trường công. Những trường tư hay chuyên biệt thì lại có mức học phí cao, không phải gia đình nào đều có điều kiện để đóng tiền.

- **Tiền trợ cấp hàng tháng:**

+ Có 3/11 thành viên trong nhóm (chị L, bác Đ, bác M) nhận được mức trợ cấp thấp nhất là 350.000 đồng/tháng vì họ khuyết tật nhẹ hơn.

+ Có 2/11 thành viên trong nhóm (Đ.Đ.K - con bác C và L.M.Q – cháu bác M) nhận được mức trợ cấp là 700.000 đồng/tháng ( do khuyết tật nặng hơn)

+ Một số thành viên khác đang làm hồ sơ xin trợ cấp, tuy nhiên thủ tục hành chính còn rườm rà nên đang gặp khó khăn chưa biết khi nào sẽ được.

**Câu 6: Những loại hình giúp đỡ nào bạn cần từ nhà nước hay người thân trong gia đình?**

- Các phụ huynh có con bị khuyết tật đang đi làm muốn cơ quan tạo điều kiện, giảm thời gian làm việc ở cơ quan để có thêm thời gian chăm sóc con ở nhà và đưa con đi khám chữa bệnh. (VD: chị H)
- Được hỗ trợ nhiều hơn về tài chính để trang trải các chi phí cho y tế, giáo dục cho con em mình.
- Tăng cường truyền thông để nâng cao nhận thức xã hội về các vấn đề liên quan đến người khuyết tật.

**Một số kiến nghị:**

- Cần có sự tác động tích cực từ phía dư luận xã hội để các nhà tuyển dụng nhân văn hơn, tạo điều kiện bố trí công việc phù hợp với khả năng của người khuyết tật và cả cộng đồng nhìn nhận cởi mở và nhân ái hơn với những người khuyết tật, không coi họ là gánh nặng mà nên nhìn nhận họ là những người có ích.
- **Quy hoạch các công trình công cộng phù hợp hơn với người khuyết tật**

+ Các trường học nên có những phòng chức năng và có giáo viên chuyên biệt để dạy cho người khuyết tật

+ Các công trình xây dựng như khu chung cư, khách sạn, nhà văn hóa… nên có lối đi dành riêng cho người khuyết tật, các cửa rộng hơn để thuận tiện cho việc đi lại của người khuyết tật.

+ Những thanh niên khuyết tật cũng có nhu cầu vui chơi giải trí nên nhà nước cần chú ý quan tâm hơn đến việc xây dựng các công trình công cộng tạo điều kiện cho người khuyết tật tiếp cận tới đó để vui chơi giải trí.

- **Xây dựng Luật và chính sách thống nhất và phù hợp với thực tiễn**

+ Luật Giáo dục quy định có 3 loại trường cho người khuyết tật là trường hòa nhập, trường bán hòa nhập và trường chuyên biệt nhưng trên thực tế người khuyết tật vẫn chưa được tham gia vào loại trường học phù hợp

+ Luật người khuyết tật có nhưng còn đưa vào thực hiện ở thực tế còn nhiều bất cập.

VD: đối với khuyết tật trí tuệ, các đặc điểm khuyết tật không biểu hiện ra bên ngoài nên trông bề ngoài họ giống như người bình thường, chỉ khi chịu tác động thời tiết hay tiếng ồn thì họ mới bị đau đầu và biểu hiện triệu chứng. Các bệnh nhân khuyết tật trí tuệ muốn được trợ cấp phải được xác nhận bị tâm thần nhưng không mấy phụ huynh muốn ghi con mình bị tâm thần.

Do vậy các cơ quan có thẩm quyền cần điều chỉnh luật và các chính sách cho phù hợp với thực tiễn.

**BÁO CÁO KẾT QUẢ PHỎNG VẤN**

**Thời gian thực hiện:** ngày 19/9/2013

**Kết quả buổi phỏng vấn:**

1. **Thông tin chung về nhóm đối tượng được phỏng vấn**

- Dạng khuyết tật: khuyết tật vận động, trong đó có 7 người khuyết tật ở chân,1 người khuyết tật cột sống, 4 người khuyết tật ở tay.
- Số lượng: tổng số có 12 đối tượng tham gia trong nhóm phỏng vấn trong đó có 5 nữ chiếm 42% và 7 nam chiếm 58%. Độ tuổi của nhóm từ 25 – 55 tuổi, trong đó ở độ tuổi từ 25 – 35 chiếm 25%, từ 35 – 45 chiếm 42 % và từ 45 -55 tuổi trở lên chiếm 33%.
- Nghề nghiệp: nghề may (3 người), làm nông nghiệp, chăn nuôi, mở cửa hàng, xây nhà trọ, trồng cây cảnh, bảo vệ, bán hàng, công nhân và có người thất nghiệp (1 người)…
- Trình độ học vấn: chủ yếu chưa tốt nghiệp phổ thông.

1. **Kết quả buổi phỏng vấn**
   1. **Khó khăn**

- **Khó khăn trong vấn đề xin việc:** Kết quả buổi phỏng vấn cho thấy chỉ có 2/12 người có nhu cầu tìm việc và họ đang ở tuổi 25 và 35. 2 người này chia sẻ họ gặp rất nhiều khó khăn trong việc xin việc. Khó khăn bởi họ không đi học nghề và cũng không đi học đại học. Do vậy, khi đi tìm 1 công việc ổn định thì họ không được tuyển dụng bởi không có nghiệp vụ chuyên môn. Một khó khăn khác mà 1 người trong nhóm chia sẻ đó là họ không có sự hỗ trợ từ phía gia đình, người thân trên cả phương diện tinh thần và vật chất, tiền bạc do vậy bản thân họ phải tự mình xoay sở. Việc tiếp cận thông tin tuyển dụng cũng không dễ dàng và họ cũng phải tự tìm đến các trung tâm giới thiệu việc làm để tìm hiểu các thông tin.

10/12 người còn lại thì tự tạo ra công việc phù hợp với đặc điểm của bản thân mình. Người thì học nghề may, người thì mở cửa hàng, người thì làm nông nghiệp, chăn nuôi, người thì bán hàng vỉa hè, người thì xây nhà trọ cho thuê, người thì trồng cây cảnh…Do vậy, đối với những người này thì không gặp khó khăn trong vấn đề tìm việc làm.

- **Khó khăn về tài chính:** 9/12 người trong nhóm phỏng vấn chia sẻ họ gặp khó khăn về tài chính. Do điều kiện sức khỏe bản thân cho nên đã ảnh hưởng tới khả năng tạo thu nhập và kinh tế của gia đình. Trường hợp của anh T. bị khuyết tật ở cột sống, rất khó khăn trong việc cử động, không làm được việc gì, gia đình lại đông người, lại phải nuôi 3 con ăn học. Một mình vợ của anh phải bươn trải để nuôi sống gia đình. Thu nhập của gia đình anh chỉ là từ làm nông nghiệp, hết thời vụ, thì vợ anh lại phải đi tìm kiếm các việc làm thuê khác để có thêm thu nhập cho gia đình. Tuy nhiên, mức thu nhập cũng rất thấp và bập bõm từ việc làm thuê.

Một trường hợp khác thì do công việc rất thất thường, lúc có lúc không. Gia đình anh có 4 người, 2 con nhỏ đang đi học. Do thu nhập không ổn định, chi phí học hành cho con cái lại rất tốn kém do đó gánh nặng về kinh tế luôn là vấn đề khó khăn nhất đối với gia đình anh.

Buổi phỏng vấn cũng cho thấy 7 trường hợp còn lại gặp khó khăn về tài cũng do hoàn cảnh của mỗi người, có người thì độc thân, tự mình phải chăm sóc bản thân, phải tự tạo thu nhập mà điều kiện sức khỏe thì lại yếu và gặp rất nhiều khó khăn trong cuộc sống hàng ngày cho nên ảnh hưởng rất nhiều tới kinh tế; có người thì phải tự mình nuôi con và bản thân…

- Kết quả phỏng vấn cũng chỉ ra rằng những người khuyết tật vận động này họ gặp **khó khăn trong việc đi lại, tiếp cận với các phương tiện giao thông công cộng**, ví dụ như việc sử dụng phương tiện là xe buýt, họ không thể tự mình lên xuống xe bus nếu như không có sự trợ giúp từ người khác. Có người thì cũng muốn tự trang bị cho mình phương tiện đi lại cá nhân để chủ động trong việc đi lại và giảm chi phí thì tìm mua phương tiện lại rất khó. Chị T. chia sẻ chị rất muốn mua 1 chiếc xe đạp điện để đi lại cho thuận tiện và cũng đỡ mệt vì hiện tại chị đi lại dùng chiếc xe đạp cũ của mình. Tuy nhiên, do chị bị khuyết tật ở tay, cho nên gặp khó khăn trong việc phanh xe nên việc mua xe cũng không thực hiện được.
- **Khó khăn trong việc tiếp cận với cơ sở hạ tầng, các công trình công cộng** thì những người tham gia phỏng vấn cũng chia sẻ họ gặp rất khó khăn, như việc đi đến các nơi công cộng, cơ quan thì rất ít nơi có đường thiết kế riêng cho người khuyết tật.
- **Thái độ của cộng đồng, gia đình đối với NKT:** Kết quả buổi phỏng vấn cho thấy sự kỳ thị của xã hội, cộng đồng đối với người khuyết tật vẫn tồn tại và không chỉ trong xã hội mà chính trong gia đình người khuyết tật cũng có sự kỳ thị đối với họ.
- **Việc tiếp cận các nguồn thông tin:** Việc tiếp cận các nguồn thông tin như các chính sách trợ cấp, các chế độ cho người khuyết tật nói chung ở mức độ vừa phải. Chưa có nhiều các hoạt động, cách thức để tuyên truyền, giới thiệu về các chương trình, chính sách hỗ trợ cho người khuyết tật. Những thông tin mà NKT có được chủ yếu là thông qua các cuộc họp của Hội NKT tại địa phương, thông qua truyền tai nhau người này bảo người kia.
  1. ***Chi phí phát sinh***

Buổi phỏng vấn cho thấy, trong cuộc sống hàng ngày của NKT có rất nhiều các chi phí phát sinh. Tuy nhiên, tập trung nhiều và phổ biến là những chi phí phát sinh sau:

- **Các chi phí y tế:** Do đặc điểm bản thân bị khuyết tật cho nên tình trạng sức khoẻ của những NKT cũng bị ảnh hưởng ít nhiều. Vì vậy, việc hay phải đi thăm khám tại các cơ sở y tế là rất thường xuyên với họ. Kết quả phỏng vấn cho thấy có 9/12 người có thẻ BHYT (chiếm 75%) và 3 người không có thẻ BHYT (chiếm 25%). Đối với những người có thẻ BHYT thì chi phí phát sinh đó là tiền khám thêm, mua thêm thuốc bên ngoài bởi trong thẻ bảo hiểm y tế thì họ chỉ được hưởng trợ cấp một số loại thuốc còn những loại thuốc khác thì họ phải tự bỏ tiền túi ra để mua. *Có trường hợp trong nhóm phỏng vấn bị bệnh ngừng thở khi ngủ, điều trị bệnh này thì lại không nằm trong diện bảo hiểm y tế, các thiết bị hỗ trợ cho việc điều trị này cũng vậy. Do vậy, họ phải chi rất nhiều tiền cho việc điều trị.*

Còn đối với những người không có thẻ BHYT thì họ phải chi trả như một người bình thường. *Chia sẻ của chị T. trong buổi phỏng vấn về trường hợp của mình. Chị không có thẻ bảo hiểm y tế, lại bị mắc bệnh thiên đầu thống. Chi phí để chị phải chi trả cho mỗi lần đi mổ là rất tốn kém và chị phải chi trả toàn bộ. Chị đã phải đi mổ đến lần thứ 4 và đã 2 năm nay chị chưa đi khám lại từ sau lần mổ cuối bởi chị sợ khi khám lại có thể chị sẽ lại phải yêu cầu mổ mà chị thì chưa có tiền.*

- Ngoài chi phí phải bỏ thêm tiền để mua thuốc ngoài bảo hiểm và trong quá trình điều trị thì những chi phí khác đi kèm khi NKT đi khám chữa bệnh đó là **chi phí cho người thân trong gia đình đi theo để giúp họ trong việc khám chữa bệnh, điều trị** (đi lại tăng thêm, ăn ở nếu phải nằm viện…)
- **Chi phí đi lại:** do là người khuyết tật vận động nên họ phải bỏ ra nhiều các chi phí cho việc đi lại bởi họ gặp rất khó khăn trong việc di chuyển: họ phải thuê xe ôm hay đi taxi để di chuyển.
  1. ***Trang trải các chi phí phát sinh***

Để trang trải các khoản chi phí phát sinh và trong cuộc sống hàng ngày thì kết quả buổi phỏng vấn cho thấy một số hướng giải quyết sau:

- **Vay vốn tín dụng:** vay vốn tín dụng để mở rộng hoạt động sản xuất kinh; để xây nhà trọ cho thuê, tạo thêm thu nhập cho gia đình;
- **Vay mượn của người thân và bạn bè:** để đóng học cho con. …..(đóng đầu năm gần 10 triệu, phải đi vay); để đi chữa bệnh.
- **Đi tìm các việc thời vụ để có thu nhập trang trải các chi phí.**
  1. ***Hỗ trợ của người thân trong gia đình***

Qua phỏng vấn cho thấy đa số tất cả những NKT trong nhóm tham gia phỏng vấn đều nhận được sự hỗ trợ của người thân. Những sự hỗ trợ này thì dưới nhiều hình thức như hỗ trợ chăm sóc cho bản thân những người khuyết tật trong cuộc sống hàng ngày, có người thì nhận được sự hỗ trợ tiền bạc của những người thân, họ hàng.

Tuy nhiên, cũng có người không nhận được sự hỗ trợ nào của người thân trong gia đình, họ phải tự mình lo hết mọi thứ từ việc chăm sóc bản thân cho đến việc tìm kế sinh nhai.

- 1. ***Hỗ trợ từ phía Nhà nước và cộng đồng***

Kết qủa phỏng vấn cho thấy NKT nhận được các sự trợ giúp từ phía Nhà nước và cộng đồng xã hội. Cụ thể, NKT nhận được những sự trợ giúp thông qua các chính sách sau:

- **Chính sách trợ cấp cho NKT:** Cuộc phỏng vấn cho thấy không phải tất cả NKT đều được nhận trợ cấp xã hội. có 8/12 NKT (chiếm 67%) được nhận trợ cấp xã hội còn 4/12 NKT không được nhận trợ cấp (chiếm 33%). NKT đánh giá cao chính sách trợ cấp của Nhà nước tuy nhiên họ cũng đề nghị là được nâng mức trợ cấp lên vì mức trợ cấp hiện tại tương đối thấp.
- **Chính sách vay vốn:** 100% những người tham gia phỏng vấn đều được vay vốn với hạn mức tối đa là 20.000.000 đồng với thời hạn là 2 năm và mức lãi suất là 0,03%/tháng, Thủ tục vay đơn giản. Họ đánh giá chính sách này rất thiết thực, hiệu quả phù hợp với nhu cầu mong muốn và khả năng chi trả của họ bởi họ chỉ phải chịu mức lãi suất rất thấp và thời gian vay cũng tương đối dài.
- **Các chính sách miễn giảm:**

Qua buổi phỏng vấn, chính sách miễn giảm của Nhà nước như đóng tiền đèn đường, vệ sinh thì có 1 người trong nhóm chia sẻ họ được hưởng chính sách này.

Chính sách miễn giảm cho NKT khi đi học: NKT đều được hưởng.

Chính sách miễn giảm cho con NKT: Qua phỏng vấn cho thấy đa số con em của NKT không nhận được bất kỳ miễn giảm nào (như giảm học phí, khi sử dụng các phương tiện công cộng…) tuy nhiên cũng qua chia sẻ của 1 người trong nhóm phỏng vấn thì con em NKT được hưởng những chế độ miễn giảm khi đi học.

- **Vé xe bus miễn phí:** Đa số NKT không sử dụng vé xe bus miễn phí bởi vì họ không sử dụng được. Việc tiếp cận sử dụng xe bus quá khó khăn đối với họ và đặc biệt có một vấn đề đó là vé xe bus ở vùng nào thì chỉ có giá trị ở vùng đó. *Một NKT trong buổi phỏng vấn chia sẻ: khi tôi đi ra tỉnh ngoài, đưa vé xe bus miễn phí ra thì anh phụ lái nói luôn rằng anh mang cái vé này về Hà Nội mà dùng, chúng tôi ở đây không dùng.* Một vấn đề khác đó là việc linh hoạt khi sử dụng vé xe bus. Ví dụ, như khi đi xe bus ở Hà Nội, nếu như NKT không đưa vé xe bus cấp cho NKT ra thì họ vẫn thu tiền như của người bình thường. Trong khi đó, ở Thành phố HCM, NKT không cần đưa vé xe bus miễn phí ra thì họ vẫn được miễn phí khi lên xe bus.
  1. **Loại hình giúp đỡ từ Chính phủ hay người thân trong gia đình:**

Kết quả phỏng vấn cho thấy NKT mong muốn nhận được sự hỗ trợ của Nhà nước trên những vấn đề sau:

- Chính sách chăm sóc sức khỏe: mong muốn Nhà nước cấp thẻ BHYT miễn phí cho tất cả NKT vì trên thực tế không phải NKT nào cũng được cấp thẻ BHYT, chỉ những người nào được nhận trợ cấp xã hội thì mới được nhận thẻ BHYT.
- Chính sách bảo trợ xã hội: Mong muốn mọi NKT đều được nhận trợ cấp xã hội bởi trên thực tế không phải NKT nào cũng được nhận trợ cấp xã hội để giảm bớt khó khăn cho NKT. Đồng thời, cũng mong muốn nâng mức trợ cấp xã hội lên.
- Các chính sách miễn giảm khác: mong muốn có chính sách miễn giảm các loại thuế giá trị gia tăng cho NKT. Ví dụ, tiền điện, nước, internet…
- Chính sách miễn giảm giá vé, giá dịch vụ khi tham gia sử dụng các dịch vụ phương tiện công cộng như tàu hỏa, máy bay; khi sử dụng dịch vụ văn hóa, thể thao, giải trí và du lịch tại các cơ sở văn hóa, thể thao: mong muốn được giảm một phần giá khi sử dụng các dịch vụ này để tạo điều kiện cho NKT hòa nhập với cộng đồng.
- Chính sách miễn giảm cho con em NKT: Qua phỏng vấn, hầu như con em NKT không được hưởng bất kỳ sự hỗ trợ hay miễn giảm nào, như học phí và các khoản đóng góp thêm ở nhà trường, tham gia các phương tiện giao thông công cộng. Gia đình họ vẫn phải đóng các khoản phí giống như con em của các gia đình bình thường. Do vậy, họ mong muốn có chính sách hỗ trợ của Nhà nước cho con em của họ.
- Công việc: Qua phỏng vấn, có một số NKT họ mong muốn có được công ăn việc làm ổn định để có thể nuôi sống bản thân, duy trì cuộc sống gia đình và giảm bớt gánh nặng cho gia đình.

**Báo cáo Nhóm Khiếm Thị 19/09**

**Về Bảo trợ Xã hội và Chính sách**

**Với Người Khuyết tật Việt Nam**

Trong khuôn khổ dự án Bảo trợ Xã hội và Chính sách với người khuyết tật Việt Nam, chúng tôi đã tiến hành thảo luận nhóm với người khiếm thính vào buổi sáng thứ 5 ngày 19 tháng 9 năm 2013 tại Trung tâm Hội người mù Huyện Thanh Trì.

Nhóm khiếm thị được phỏng vấn bao gồm 9 người, bao gồm 3 nam và 6 nữ khiếm thị. Tổng thời gian làm việc là 2 tiếng đồng hồ tính từ thời điểm tập hợp nhóm lúc 9h00, bắt đầu thảo luận lúc 9h30, và kết thúc vào lúc 11h10.

Hình thức thảo luận của nhóm là người phỏng vấn đưa ra 6 câu hỏi lớn và khuyến khích các thành viên trong nhóm khiếm thị tự chia sẻ các thông tin liên quan. Trong suốt buổi thảo luận, các thành viên đã nhiệt tình chia sẻ quan điểm cá nhân, các vấn đề được đề cập, cũng như nêu lên một số mong muốn của bản thân.

Độ tuổi trung bình của nhóm là 30 – 50 với đa số thành viên đã lập gia đình và đang làm công việc tẩm quất tại hội người mù. Trẻ nhất trong nhóm là 24 tuổi trong khi người lớn tuổi nhất là 61 tuổi, đã có 13 năm làm việc tại hội người mù.

Khi được hỏi về ***những khó khăn khi xin việc*** *(câu 1)*, người mù gần như không có nhiều cơ hội để làm những công việc của người sáng mắt. Những công việc như làm tăm tre hay tẩm quất cần họ phải tập trung trong một hội để có được sự giúp đỡ nhất định chứ tự bản thân tìm việc là rất khó khăn. Đây cũng có thể là một đặc thù của người khiếm thị, họ thường chỉ làm một vài công việc cụ thể, điển hình nhất là công việc tẩm quất với đa số thành viên trong nhóm đang làm. Một số thành viên khác không có khả năng xin việc cũng như đòi hỏi của gia đình cần phải chăm sóc con cái nên họ ở nhà làm công việc nội trợ.

Những thành viên trong nhóm, trong đó có chú T. cho rằng người mù là nhóm khuyết tật thiệt thòi nhất vì việc không nhìn được đã tước đi cơ hội làm đa số việc công việc trong xã hội. Bên cạnh đó, ngay cả khi người mù có chuyên môn hoặc có tay nghề, nhưng các dây chuyền máy móc hay cơ sở vật chất lại không đáp ứng được những đòi hỏi đặc biệt từ cá nhân người mù. Vì hoàn cảnh đặc biệt, những máy móc hay cơ sở vật chất này cần phải được điều chỉnh để phù hợp nhưng nếu việc lắp máy móc điều khiển bằng giọng nói ở thang máy là có khả thi thì mỗi lần các nhà máy thay đổi dây chuyền sản xuất là một lần người mù sẽ phải học để làm quen lại từ đầu. Thế nên, theo chú T., cơ quan người ta không nói nhưng bản thân nhân viên người khiếm thị đã tự ra đi. Đây là một rào cản rất lớn với toàn bộ các thành viên trong nhóm.

Khi đề cập tới vấn đề tự tin, các thành viên cho rằng tuy sự cạnh tranh từ các những người sáng mắt là rất cao nhưng một khi đã quyết định bước chân ra ngoài xã hội, những người khiếm thị đã rất tự tin vào bản thân mình. Cái khó là các nhà tuyển dụng không thật sự muốn thu nhận người mù vì chỉ có một số công việc phù hợp với họ và nếu được thu nhận, người mù vẫn phải vượt qua một thử thách nữa, đó là sự đối xử không công bằng khi các nhân viên sáng mắt được tăng lương hay khen thưởng nhưng người mù lại không được. Cùng một công việc, có khi người mù làm tốt hơn nhưng sự ghi nhận từ công ty lại không công bằng. Áp lực như vậy khiến người mù thấy tự ti và mặc cảm.

Vấn đề đi lại là một vấn đề cơ bản, đa số mọi người chọn đi lại bằng xe buýt và được hỗ trợ rất nhiều từ xã hội từ thái độ giúp đỡ của những người cùng đi xe hay sự cởi mở của các tài xế, tuy nhiên khi chỉ có một thân một mình thì việc bắt đúng xe buýt là việc gần như không thể. Chưa kể ngày mưa thì việc đi làm lại càng thêm khó khăn. Cây gậy dùng để dẫn đường thì phải thay thường xuyên và đôi khi lạị bị một số người xấu trêu chọc lấy mất gậy. Con số này là không đáng kể nhưng thực sự là có. Nếu các xe buýt có loa báo, các nhà cao tầng có máy ghi âm phần hướng dẫn sơ đồ, một số thành viên cho rằng việc họ đi làm sẽ đỡ vất vả hơn.

Về trình độ văn hóa, người khiếm thị thực sự cần các chương trình đào tạo nghề hay chứng chỉ để họ có thể tham gia hành nghề ngoài xã hội cũng như là được định hướng xem họ có thể làm được việc gì. Nhiều người khiếm thị không có sự hòa đồng với xã hội nên cần phải có hội tham gia công tác tư vấn để thuyết phục gia đình họ và bản thân họ dám đi làm ngoài xã hội. Người khiếm thị cũng có cơ hội học chữ nổi nhưng chỉ giải quyết được nhu cầu cá nhân còn để dùng trong thực tế thì có thể nói là không thể.

Vấn đề tài chính, theo anh L. thì riêng chuyện học cái gì đã khó nhưng khi biết là cần học cái gì thì việc có đủ tiền hay không lại là một câu chuyện khác. Ví dụ như chứng chỉ tẩm quất là 3 triệu nên không phải ai cũng dám học để lấy chứng chỉ.

Thái độ gia đình của đa số thành viên là rất tích cực, tuy nhiên theo cô C. thì không phải ai cũng đồng cảm hay chia sẻ. Và theo chú L., khi đã có thu nhập thì tiếng nói của người khuyết tật sẽ thay đổi. Bản thân các thành viên đã đến vận động để gia đình nhiều người khiếm thị đồng ý cho người thân của họ được đi làm. Không tin tưởng vào khả năng tự lập là tư tưởng phổ biến, nhưng nếu chiều chuộng quá lại không hay.

Trong quá trình trao đổi, anh Đ. cho biết nếu người mù có thể sử dụng máy vi tính thì cơ hội việc làm là không ít nhưng không phải ai cũng có điều kiện như vậy. Hội người mù là cơ sở cung cấp thông tin việc làm hay cho vay vốn tốt nhất và uy tín nhất cũng vì lý do như vậy.

Về các ***chi phí phát sinh*** *(câu 2)*, khoản chi phí về y tế là quan trọng nhất. Thu nhập ít, loại hình công việc không nhiều nhưng khoản tiền phải chi cho các loại thuốc những khi mắt bị đau nhức lại không hề nhỏ. Bảo hiểm y tế tuy đã chi trả một phần nhưng có nhiều loại thuốc lại không được liệt vào loại giảm giá cũng như là tổng số tiền mà người khuyết tật phải chi trả vẫn rất nhiều so với thu nhập chung.

Chi phí tiếp theo là chi phí cho gậy dẫn đường. Người mù thường phải mua các loại gậy này giá 120,000 - 150,000VND và phải thay sau khoảng 6 tháng. Các thiết bị như vậy là cần thiết vì đi ra đường là cần dùng gậy. Bên cạnh đó, các loại di động đặc biệt cũng là một khoản chi phí phát sinh khác. Những loại điện thoại này phải được thiết kế riêng biệt nên không dễ dàng để người mù tìm mua và có đủ tiền để mua.

Có một chi phí khác nữa hơi đặc thù, đó là sách chữ nổi hay băng ghi âm đều rất đắt. Nếu không được hỗ trợ, với tình hình tài chính của người khiếm thị họ sẽ hoàn toàn không có cơ hội để tiếp cận.

Khi được hỏi về việc làm thế nào để ***trang trải các chi phí sinh hoạt hàng ngày*** *(câu 3)* cũng như các chi phí phát sinh, một câu trả lời đơn giản là hãy tiết kiệm và lên kế hoạch chi tiêu một cách hợp lý. Các thành viên đều cho rằng bản thân người mù phải tự lập nên việc phụ thuộc vào người khác là không nên. Chuyện vay mượn là có nhưng họ cần tự chủ. Qua đó cũng có thể thấy nhu cầu việc làm để tự lập quan trọng tới mức nào với người mù.

Với câu hỏi về ***sự giúp đỡ của người thân trong gia đình*** *(câu 4)*, việc đi lại, đã được đề cập một phần bên trên, người thân sẽ hỗ trợ rất lớn người khiếm thị đặc biệt là thời gian đầu làm quen với việc ra đường. Chuyện dạy dỗ con cái thì đặc biệt người mù phải nhờ sự giúp đỡ của mọi người vì không phải ai cũng được học qua trường lớp đầy đủ hơn nữa họ không nhìn thấy gì để dạy con cả nên quá trình theo dõi tình hình học tập của con cái là rất khó khăn.

***Sự giúp đỡ của chi hội*** *(câu 5)* có thể xem là cứu cánh lớn nhất của người mù. Thứ nhất, thông qua hội những người khiếm thị tiếp cận được các thông tin về chính sách của chính phủ. Thứ hai, cơ quan hội cũng đứng ra bảo lãnh để các thành viên hội được vay vốn hoặc trực tiếp tạo công ăn việc làm. Chú L. đã chia sẻ, *“Không có hội, ai dám cho người mù vay, dù là 1 triệu?”* Chính quyền địa phương cũng thông qua hội để nắm tình hình người khiếm thị và cũng nhờ đó mà các tổ chức trong nước và quốc tế có thể tiếp cận để giúp đỡ và đưa ra những chương trình hành động cụ thể.

Bản thân chi hội cũng nhận được sự giúp đỡ từ cộng đồng và chính quyền. Cơ sở của hội khang trang là nơi sinh hoạt và làm việc của không chỉ hội người mù. Cơ sở này, thuộc huyện Thanh Trì, có lẽ là cơ sở tốt nhất trong số các cơ sở của người mù.

Chính phủ nhìn chung có nhiều hỗ trợ với người khuyết tật nói chung và người mù nói riêng trong đó có khoản hỗ trợ vay vốn và việc cấp thẻ bảo hiểm y tế miễn phí, các thành viên đều nhất trí rằng những sự trợ giúp này rất hữu hiệu. Con cái của các thành viên cũng được miễn giảm học phí hay có nhiều sự hỗ trợ từ nhà trường và chính quyền. Ngoài ra, các hoạt động văn hóa - thể thao cũng được chính quyền quan tâm và khuyến khích.

Nói về mong muốn của người khiếm thị, các thành viên cho rằng trợ cấp tiền mặt là đáng quý nhưng một chính sách hỗ trợ thiết thực sẽ có tính hiệu quả lâu dài hơn. Ví dụ như đưa ra các chính sách cụ thể về hỗ trợ, tìm kiếm những nghề phù hợp để người khiếm thị có việc làm ổn định. Bảo hiểm y tế nếu có thể thì có các khoản chi trả cao hơn miễn phí cho người mù, nếu ai có hoàn cảnh khó khăn hơn thì được hỗ trợ nhiều hơn. Việc ưu tiên khi đi khám bệnh hay tại nhiều nơi công cộng khác cần có sự ưu tiên nếu không sẽ rất thiệt thòi cho người khuyết tật. Các phương tiện đi lại có thể được miễn phí hoặc hỗ trợ, ví dụ như tại các điểm xe buýt có thể có vị trí riêng cho người mù lên xe hay các hàng hãng không có chương trình vé riêng cho người mù vì khi họ nghe tin có vé giảm giá thì thường là đã hết vé.

Nhiều thành viên đồng ý về vai trò của Nhà nước trong việc làm thay đổi nhận thức của cộng đồng. Ví dụ như Nhà nước có thể mời những gương điển hình của người khuyết tật tham gia các chương trình trao đổi trên ti vi cho cộng đồng thấy người khuyết tật không phải gánh nặng, họ tự lập và có ý chí phấn đấu.

**BÁO CÁO KẾT QUẢ PHỎNG VẤN**

*Về “Bảo trợ xã hội và chính sách với người khuyết tật Việt Nam” được tiến hành tại:*

- Địa điểm: Hội trường Hội người khuyết tật Huyện Thanh Trì, Hà Nội
- Thời gian: 9g00 – 11g00, thứ Năm ngày 19 tháng 9 năm 2013.

Số lượng: Có 07 người (6 nữ, 1nam)

Độ tuổi của nhóm: từ 45 – 65

Nghề nghiệp; Phần lớn là ở nhà chăm sóc con, công nhân, nội trợ, thợ may, hội.

**Nội dung buổi phỏng vấn tập trung trả lời 6 câu hỏi sau:**

**Câu 1: Người khuyết tật thường gặp nhiều khó khăn trong vấn đề xin việc làm. Anh/ chị đã từng gặp phải những khó khăn đó như thế nào?**

Qua thảo luận thì phần lớn các thành viên trong nhóm đều có ý kiến là con cái họ đang và sẽ gặp

- **Khó khăn tìm kiếm việc làm**: Do phần lớn con họ bị khuyết tât nặng. Trong số con của các thành viên được phỏng vấn, 2 người bị liệt toàn phần (con của chị Vân và anh Tươi).
- Di chuyển, phần lớn con họ không chủ động đi lại mà tất cả đều phụ thuộc vào người nhà nên để có việc làm cũng khó khăn
- Khó khăn về trình độ văn hóa: Phần lớn không có điều kiện đến trường, một số trường không nhận trẻ khuyết tật (con họ đều gặp vấn đề về nhận thức) 5/7.
- Riêng chị H. bị khuyết tật vận động, hiện đang đang là chủ tịch Hội người khuyết tật huyện Thanh Trì. Chị làm thợ may tại nhà
- Chị VHY (con chị V.) đã tốt nghiệp Trung cấp về Công nghệ thông tin. Nhưng hiện nay cũng chưa có việc làm, tuy đã nộp hồ sơ xin việc nhiều nơi.
- Khó khăn về giao tiếp cũng là 1 trở ngại lớn đối với con cái họ.
- **Thái độ của gia đình:** Hầu hết các bậc phụ huynh đều xác định là chăm sóc và dạy cho con cái họ biết cách tự chăm sóc biết vệ sinh cá nhân cho tốt để con có thể sinh hoạt tự lập là tốt lắm rồi, mà không hy vọng con của họ có khả năng lao động. Theo bác T, (Hội nạn nhân chất độc da cam) cho biết “có tới 70% nạn nhân trực tiếp và 30% nạn nhân có con bị ảnh hưởng từ bố mẹ tham gia chiến tranh, người thấp tuổi nhất trong hội là 62 tuổi… do ảnh hưởng về tâm lý, sức khỏe cũng như hoạt động nên việc đi học nghề và xin việc là rất khó”, cá biệt trong hội chỉ có 2 cháu là con của hội viên là có khả năng lao động và xin việc.
- **Thái độ phân biệt đối xử của nhà tuyển dụng**: Ví như trường hợp của chị V chia sẻ: “*Tôi đã ủng hộ tích cực tính ham học và chịu khó vươn lên của con. Từ lớp 1 đến lớp 5 tôi xin cho con học ở Hòa Bình. Lên cấp 2, đầu tiên giáo viên chủ nhiệm không dám nhận nhưng sau đó tôi làm giấy cam đoan và nộp học bạ để cho con đi học. Thi vào cấp 3 con tôi không đủ điểm nhưng sau đó tôi vẫn cố gắng tạo điều kiện cho con đi học trung cấp về tin học và cháu đã tốt nghiệp bằng trung bình. Tuy nhiên khi đi xin việc, nhà tuyển dụng chỉ động viên chứ không nhận vào làm việc”*

**Câu 2: Trong gia đình của những người khuyết tật thường có những khoản chi phí phát sinh. Với cá nhân bạn những chi phí đó là gì?**

Qua thảo luận 100% các thành viên của nhóm đều cho rằng chi phí phát sinh lớn nhất là;

- **Chi phí y tế**

Cụ thể như:

+ Chị U: Trong não con chị có di chứng do sốt giật từ khi 8 tháng tuổi. Thường chị phải chi trả tiền thuốc men cho cháu là 600.000 đồng/ tháng và mỗi lần đi khám là 100.000 đồng.

+ Chị V: chi phí thuốc men và xoa bóp cho con chị phải trả khoảng hơn 1 triệu đồng/ tháng

+ Chị X: con chị yếu, sức đề kháng kém nên bệnh triền miên nên phải uống nhiều thuốc kháng sinh, trung bình chị phải chi hơn 1 triệu/tháng. Riêng thuốc bổ não giá khoảng 2 triệu/lần nên thỉnh thoảng chị mới mua được cho con dung.

+ Bác T: phải chi khoảng 2.700.000 – 3.000.000 đồng/tháng cho thuốc men và vật lý trị liệu cho con. Hàng tháng bác nhờ bác sỹ ở bệnh viện Thường Tín chăm sóc, giúp con có nhận thức vệ sinh để gia đình đỡ vất vả.

- **Chi phí giáo dục:**

Chị H: phải nghỉ việc làm để ở nhà chăm sóc và dạy dỗ con nhưng chị vẫn thuê cô giáo về nhà dạy ngôn ngữ và cách tự vệ sinh cho cháu, 1 tiếng/ ngày với mức phí 200.000 đồng/ tiếng. Trung bình mỗi tháng tính cả chi phí thuốc men, bấm huyệt và tiền học của con chị là khoảng 3 triệu/ tháng.

**Câu 3: Làm thế nào anh/ chị trang trải được những chi phí này?**

Phần lớn họ cho ý kiến là: Mọi chi phí về y tế, giáo dục hay đi lại chủ yếu do gia đình tự chi trả

+ Chị U: “*vợ chồng tôi nói chung là tự trả thôi chứ cũng không có hỗ trợ từ ngoài gì cả”*

+ Chị V: *“Tôi làm công nhân thì cứ tiết kiệm để chi trả hàng tháng cho con”*

+ Bác T: *“Tôi được hưởng chế độ bệnh binh, vợ tôi làm nông nghiệp, cũng yếu rồi, các chi phí chỉ do mình tôi tằn tiện để lo cho cháu”*

**Câu 4: Các thành viên khác trong gia đình đã hỗ trợ thành viên khuyết tật trong gia đình như thế nào?**

Phần lớn các thành viên trong nhóm đều nhận được sự giúp đỡ, hỗ trợ từ bố mẹ, anh chị em và người thân trong gia đình về tài chính như:

- Tiền chi trả cho thuốc men, khám bệnh, giáo dục.
- Hầu như các mẹ có con khuyết tật nặng đều không đi làm để ở nhà tự chăm sóc con, như chị H, chị X. nói *“khi phát hiện cháu bị tự kỷ từ năm 3 tuổi, tôi đã bỏ việc để ở nhà chăm con cho chu đáo”*

**Câu 5: Những khoản hỗ trợ nào mà các anh/ chị đã nhận được từ nhà nước hay từ các thành viên khác trong gia đình? Anh/ chị đánh giá thế nào về những sự hỗ trợ đó?**

Có 5/7 % trong nhóm đều nhận được tiền trợ cấp hàng tháng của nhà nước, 2/7 chưa nhận được vì đang làm thủ tục xin trợ cấp. Các mức trợ cấp khác nhau, do tình trạng khuyết tật khác nhau. Riêng chế độ trợ cấp cho nạn nhân chất độc da cam thường cao hơn người khuyết tật khác.

+ Bác T: nhận được chế độ chất độc da cam là 1.100.000 đồng/ tháng và đã nhận được chế độ này khoảng 10 năm.

+ Chị V: nhận được chế độ chất độc da cam là 700.000 đồng/ tháng, mới được 1 năm nay.

+ Chị H: nhận được hỗ trợ con khuyết tật là 525.000 đồng/ tháng

+ Chị X: đang làm thủ tục xin trợ cấp, nhìn chung thủ tục khá rườm rà và phức tạp

+ Chị V: được hỗ trợ cho ngườ khuyết tật là 350.000 đồng/ tháng

- Quà tặng của chính quyền địa phương trong dịp lễ, tết

Chị V: “*khi con tôi chưa đến 16 tuổi thì gia đình chị có nhận được quà từ huyện nhưng khi cháu được 16 tuổi thì không được phát quà nữa”.*

Chị V: vào Ngày dành cho người khuyết tật và các dịp lễ Tết vẫn nhận được quà, ngoài ra còn được Hội chữ thập đỏ tặng xe lăn.

- Giáo dục:

Chị X: nhờ xin được chế độ cựu chiến binh ở xã nên nay con chị được đi học miễn phí, nếu không, như trước đây mức học phí chị phải đóng là 4 triệu/ tháng, chị cho con đi học được một năm thì hết tiền, phải tạm đưa con về quê.

Phần lớn các thành viên được phỏng vấn cho biết, họ thường nhận được sự hỗ trợ từ người thân trong gia đình (ông bà ngoại, ông bà nội, các cậu, các chú)

- Ngoài ra còn có sự giúp đỡ của các nhà tài trợ:

Theo bác T, khi vận động được các nhà tài trợ thì cũng có quà, chia phát cho các thành viên trong Hội để hỗ trợ, động viên tinh thần lẫn nhau.

- Sự hỗ trợ của cơ quan

Chị V: khi con còn bé thì nhờ bà ngoại chăm sóc, ngoài ra cũng được cơ quan tạo điều kiện về thời gian để chăm sóc con.

Nhìn chung các thành viên tham gia phỏng vấn đều nhận thấy các chính sách của nhà nước hiện nay có quan tâm hơn đến người khuyết tật và thấy đỡ khó khăn hơn khi có trợ cấp.

**Câu 6: Những loại hình nào các bạn cần từ nhà nước hay người thân trong gia đình?**

Có 100% thành viên mong muốn:

- Cần nhất là nhà nước cung cấp những công việc phù hợp cho người khuyết tật.
- Trợ cấp tiền mặt từ Nhà nước cao hơn để gia đình đỡ khó khăn như: Chị Xuân: vì con chị cũng không làm được gì nên cũng mong nhà nước cho cháu một khoản tiền để đỡ gia đình, khoảng 1 triệu/ tháng.
- Các phụ huynh cũng mong con được đi học.
- Thay đổi nhận thức xã hội về người khuyết tật: Chi U nói: “*cộng đồng, hàng xóm có ác cảm, có người nhìn thấy con tôi thì dắt con mình đi ra chỗ khác, nhưng cũng có người động viên an ủi và chia sẻ cảm thông”.* Chị X: “*định kiến bên nhà chồng, chị em dâu thì thào còn mẹ chồng thì bảo “thằng dở hơi nó về nhà tôi”*

Nhưng các chị đều hài lòng nói tốt về chồng mình vì các anh tốt và thương con nên động viên các chị chịu khó chăm con.

Nhìn chung, mọi người đều rất cởi mở chia sẻ những khó khăn, vất vả của bản thân và gia đình khi có con bị khuyết tật. Ai cũng mong nhận được sự hỗ trợ y tế để giúp cho con họ được chăm sóc, phục hồi sức khỏe tốt hơn, biết tự phục vụ cho cá nhân để có thể sống tự lập được tốt nhất.
